# Genome-wide transcriptional profiling and physiological investigation elucidating the molecular mechanism of multiple abiotic stress response in *Stevia rebaudiana* Bertoni

**DOI:** 10.1038/s41598-023-46000-7

**Published:** 2023-11-13

**Authors:** Poonam Pal, Mamta Masand, Shikha Sharma, Romit Seth, Gopal Singh, Sanatsujat Singh, Ashok Kumar, Ram Kumar Sharma

**Affiliations:** 1 Biotechnology Division, CSIR-Institute of Himalayan Bioresource Technology (CSIR-IHBT), Palampur-176061, India; 2 Academy of Scientific and Innovative Research (AcSIR), Ghaziabad-201002, India

**Keywords:** Biochemistry, Biotechnology, Computational biology and bioinformatics, Genetics, Molecular biology, Physiology, Plant sciences

## Abstract

Considering the major source of plant-derived low/non-calorie steviol glycosides (SGs), comprehensive physiological, biochemical, and deep transcriptional investigations were conducted to explicit deeper insight into multiple abiotic stress responses in *Stevia rebaudiana*. The physiological indicators including photosynthesis, chlorophyll, relative water content, shoot growth, electrolyte leakage, and SG biosynthesis were negatively impacted under drought (DS), followed by salinity (SS) and waterlogging (WS). Global transcriptional analysis revealed significant upregulated expression of the genes encoding for ROS detoxification (GST, SOD, APX, glutathione peroxidase), osmotic adjustment (alpha-trehalose-phosphate and S-adenosylmethionine decarboxylase), ion transporters (CAX, NHX, CNGS, VPPase, VATPase), water channel (PIP1, TIP) and abiotic stress-responsive candidate genes (LEA, HSPs, and Dehydrins) regulating abiotic stress response in *S. rebaudiana*. These inferences were complemented with predicted interactome network that revealed regulation of energy metabolism by key stress-responsive genes (GST, HKT1, MAPKs, P5CSs, PIP), transcription factors (HSFA2, DREB1A, DREB2A), and abiotic stress responsive pathways (ABA, ethylene, ion stress). This is the first detailed study to comprehend the molecular regulation of stress response and their interplay under DS, SS, and WS. The key genes and regulators can be functionally validated, and will facilitate targeted gene editing for genetic improvement of crop sustainability under changing environmental conditions in *S. rebaudiana.*

## Introduction

*Stevia rebaudiana* Bert. (Family: Asteraceae) is a source of plant-derived low/non-calorie natural sweeteners (LNCSs) known as ‘Steviol glycosides (SGs)’. It is widely cultivated in various countries in South & North America, Asia, and Europe^1^. So far, more than 60 different kinds of SGs including Rebaudioside-A (Reb-A), Rebaudioside-D (Reb-D), and Rebaudioside-M (Reb-M) with high sweetening properties and the least bitter aftertaste effect have been discovered (87th JECFA report, 2019: http://www.fao.org/3/ CA0953EN/ca0953en.pdf^2^). Based on their proportion in leaf dry weight, SGs have been categorized into major [Stevioside (Stev): 5–10%; Reb-A: 2–4%] and minor [Reb-D and Reb-M (0.4–0.5%)]^3^. Desirable attributes including 300 times greater sweetening properties than table sugar, and safe metabolization in the human digestive system make SGs a better replacement for both artificial low-calorie and conventional high-calorie sweeteners^2^. Considering wider popularity in the commercial market, and to cater with climate change issues, the existing biochemical and physiological inferences of abiotic stress vulnerability require an in-depth understanding of underlying molecular mechanism in *S. rebaudiana*. Global climate change with exposure of severe environmental conditions has a major negative impact on plant growth and development. It is estimated that over 51–82% of annual crop loss is caused by incidence of multiple abiotic stresses, worldwide^4^. Among these, drought, salinity, temperature, and waterlogging are major factors affecting crop yield and productivity, significantly^5^. It is predicted that drought stress severity alone may cause a change in the growth pattern by reducing nearly 50% of global crop production^6^. Similarly, 100 and 200 mM of NaCl have exhibited with growth arrest or decreased crop yield in glycophytes including major crop plants (*Triticum aestivum, Oryza sativa*), then the halophyte (*Suaed arigida*) with the ability to tolerate 300–500 mM of NaCl^7,8^. Likewise, waterlogging stress resulted in a 20–50% loss in global wheat production^9^. Therefore, it is important to understand insights into multiple stresses which can help for developing stress resilient crop varieties that are better ability to tolerate/ adapt these stressors, ultimately improving food security.

The response of plants to abiotic stress is a multifaceted mechanism that entails stress perception in both leaf and root tissue, initiating a cascade of signaling processes and transcription factors that regulate primary metabolic processes, stress-responsive pathways, and genes^10–12^. Molecular-level modulations orchestrated by transcription factors including AP2/ERF that regulate plant hormones (ABA, ET, GAs, and CTK) through intricate feedback mechanisms, while PIF4/PIF5 play a crucial role in shoot branching. Likewise, upregulated expression of nuclear factor Y (NF-Y) family improved performance under drought stress via elevated photosynthesis rates. This intricate network leads to maintain plant biomass and adaptability under abiotic stress^13–15^. Water availability (drought, waterlogging), or quality (salinity), significantly disrupts metabolic processes by causing osmotic or ionic imbalances, oxidative damage, alterations in stomatal conductance, photosynthesis, tissue water potential, ABA biosynthesis and cellular membrane integrity^16,17^. The analysis of abiotic stress in *S. rebaudiana* has shown little effect on plant growth and other physiochemical processes under mild/short-term stress conditions, while highly impacted under severe/long-term stress conditions^18–20^. The mild salt stress has resulted in an incremental accumulation of SGs^15^. Although these studies have shed light on the physiological and biochemical responses to abiotic stress, however, our understanding of its adaptive responses remains limited. Therefore, deeper insight into the underlying molecular mechanisms implicated in multiple abiotic stress responses needs to be investigated in *S. rebaudiana.*

The next-generation sequencing (NGS)-assisted transcriptional analysis has been successfully applied for elucidating multiple stress-responsive global gene expressions, pathways, genes, and regulators, including biosynthesis of bioactive metabolite biosynthesis in various plant species, which is yet to be understood in *S. rebaudiana*^18,21,22^. Therefore, the current study was carried out to unravel the morpho-physiological and transcriptional insights during drought (DS), salt (SS), and waterlogging (WS), and its influence on steviol glycoside biosynthesis. Subsequently, a complex gene network was predicted which identified key regulatory genes including transcription factors and transporters along with pathways influencing plant adaptability and SG biosynthesis. The key genes and regulators identified in this study can be functionally validated, and potentially utilized for genetic manipulations for development of abiotic stress-resilient high yielding Stevia cultivars that can allow the plant to withstand under adverse environmental conditions.

## Results

### Abiotic stress-mediated morpho-physiological variations

**Figure 1:**
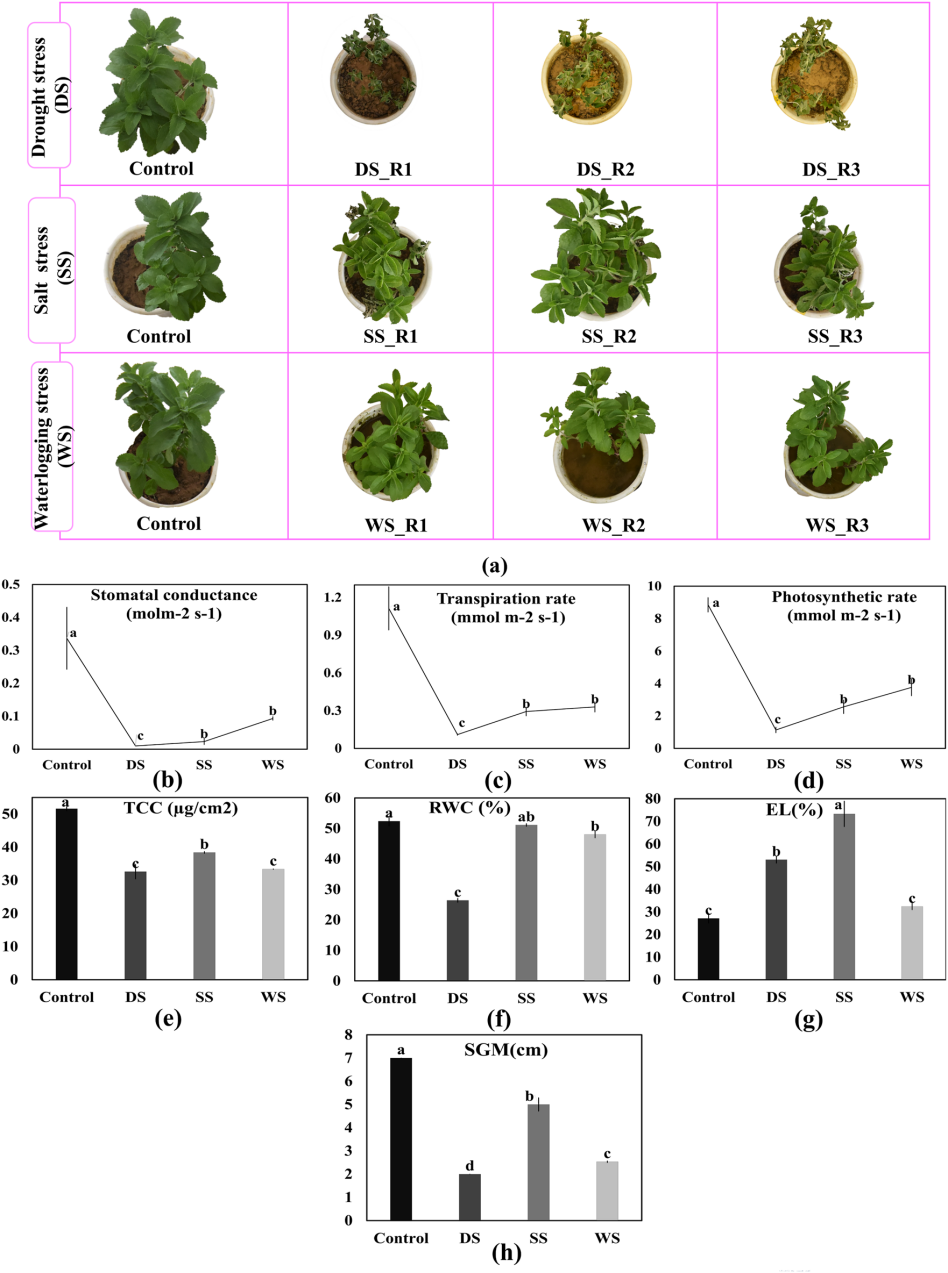
Morpho-physiological response of treated and control plants under DS, SS and WS after 30 days of treatment in *S. rebaudiana*. (**a**) Morphological response in three biological replicates under drought (DS_R1, DS_R2, DS_R3), salinity (SS_R1, SS_R2, SS_R3) and waterlogging stress (WS_R1,WS_R2,WS_R3); (**b**) Stomatal conductance(Gs); (**c**) Transpiration rate(E); (**d**) Photosynthetic rate(Pn); (**e**) Total chlorophyll content(TCC); (**f**) Relative water content(RWC); **(g)** Electrolyte leakage(EL); (**h**) Shoot growth measurement (SGM). Data are presented as the mean of three biological replicates (± SD). Different letters indicate significant differences (*P* < 0.05) according to Duncan’s test.

The morpho-physiological analysis exhibited with significant variations under three abiotic stress treatments (DS, SS, and WS) in comparison to control (C) samples after 30 days of treatments (Fig. 1a). A significant decrease in gaseous exchange (Gs) affecting photosynthesis per unit area (Pn) and transpiration rate (E) was recorded under DS (0.01molm-2 s-1), followed by SS (0.02molm-2 s-1) and WS (0.10molm-2 s-1) as compared to control (0.33molm-2 s-1) (Fig. 1b). The transpiration (E) and photosynthesis rate (Pn) were corresponded with the Gs trend, however with no significant difference in E and Pn under SS and WS (Fig. 1c,d). However, total chlorophyll content (TCC) recorded with significant reduction under DS (32.6 µg/cm^2^), SS (38.4 µg/cm^2^), and WS (33.3 µg/cm^2^) (Fig. 1e). Further, a considerable reductions (26%) in relative water content (RWC) was recorded under DS than WS, while, remained nonsignificant under SS (Fig. 1f). An increasing trend of electrolyte leakage (EL) was observed under DS (26%) and SS (46%) with nonsignificant change under WS (Fig. 1g).

### Effect of abiotic stress on Stevia's growth

Shoot growth exhibited with substantial difference between treated (DS, SS, and WS) and control (C) plant samples. Wherein, overall growth exhibited with up to 70% reduction under DS and WS, while 28% reduction was recorded under SS that with control plants (Fig. 1h).

### Analysis of Steviol glycosides (SGs) content

**Figure 2:**
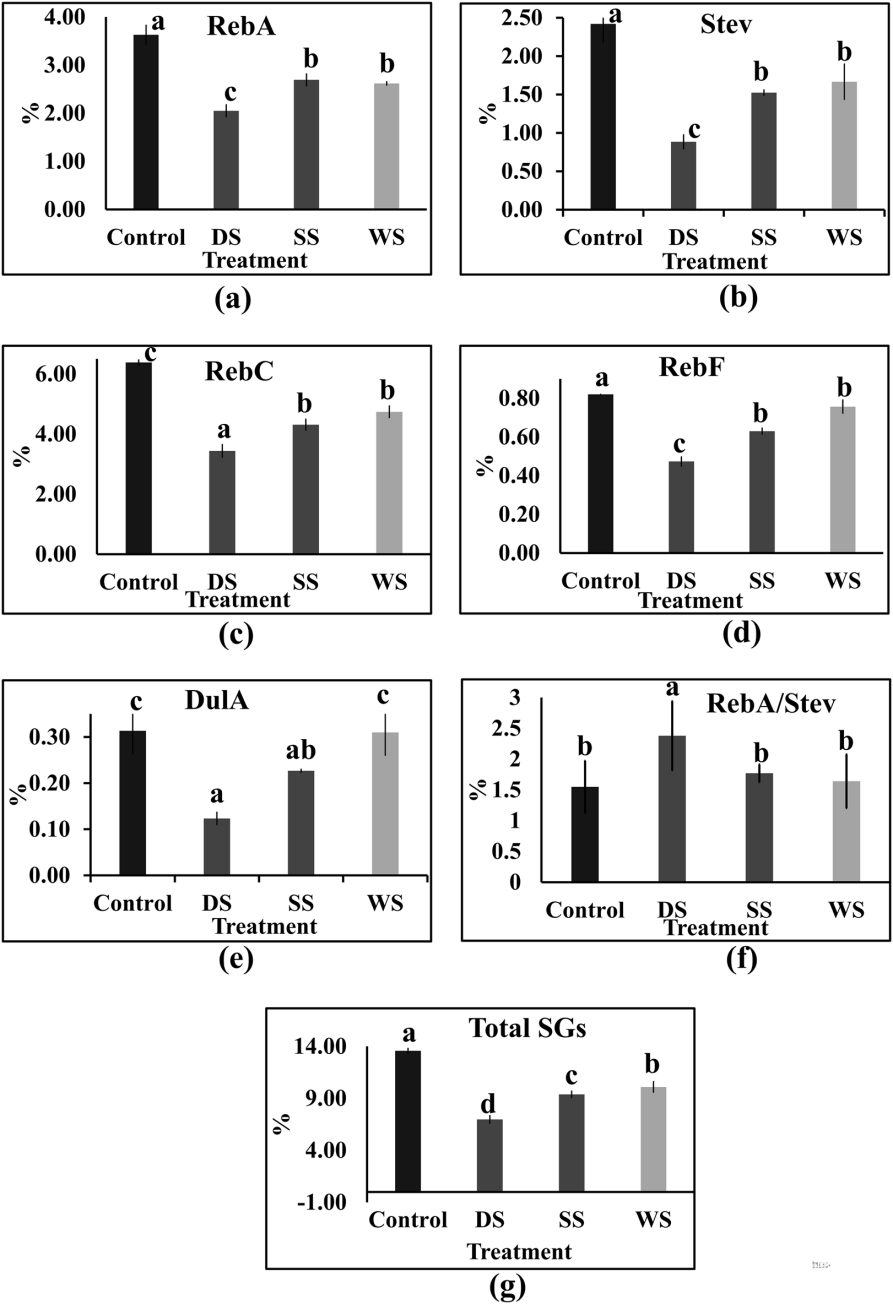
Biochemical estimation of different components of SGs in plants treated with abiotic stress. (**a**) Rebaudioside A(RebA); (**b**) Stevioside (Stev); (**c**) Rebaudioside C (RebC); (**d**) Rebaudioside F (RebF); (**e**) Dulcoside A (DulA); (**f**) RebA/Stev; (**g**) Total SGs. Data are presented as the mean of three biological replicates (± SD). Different letters indicate significant differences (*P* < 0.05) according to Duncan’s test.

Analysis of total SGs and five components SGs (RebA, Stev, RebF, RebC, and DulA) recorded with decline trend under DS, SS, and WS (Fig. 2). Total SGs content was recorded with substantial reduction irrespective of three abiotic stresses with severe impact under DS (7%) than SS (4%) and WS (3%) in comparison to control (Fig. 2g). While, SGs composition (RebA/Stev ratio) was also recorded with variable incremental change under DS treatments than the control sample **(**Fig. 2f). Interestingly, earlier studies have indicated the role of RebA in an osmotic adjustment under abiotic stress influencing compositional changes in SGs accumulation^23^.

### Transcriptome sequencing and functional annotation

Illumina-Novaseq sequencing of 24 cDNA libraries representing treated (DS, SS, WS) and control samples resulted in ~ 41 billion raw reads (Table ST1). More than 30 billion filtered reads were obtained in quality filtering suggesting sample-wise deep coverage of RNA seq data (Table ST1). *De-novo* assembly resulted into 318,859 non-redundant (NR) transcripts with an N50 and average contig length of 1396 bp and 861 bp, respectively (Table ST2). Further, reference-based mapping to the chromosome-level genome assembly resulted in an average mapping of 83.66% with 241,653 contigs successfully mapped to the public genome of *S. rebaudiana* (Table ST1). Interestingly, 75% of the NR transcripts were functionally annotated to public data bases including NCBI-nr (254,875), KEGG (20,607), Swiss-prot (162,065), TAIR (143,140), and plant TF (71,297) database suggests the potential utility of current data for deeper understanding of regulatory mechanism of DS, SS and WS (Figure S1a; Supplementary data 1). All the raw reads were submitted to the National Centre for Biotechnology Information (NCBI) Sequence Read Archive (SRA) under the bio project PRJNA909184.

### Differential gene expression analysis

**Figure 3:**
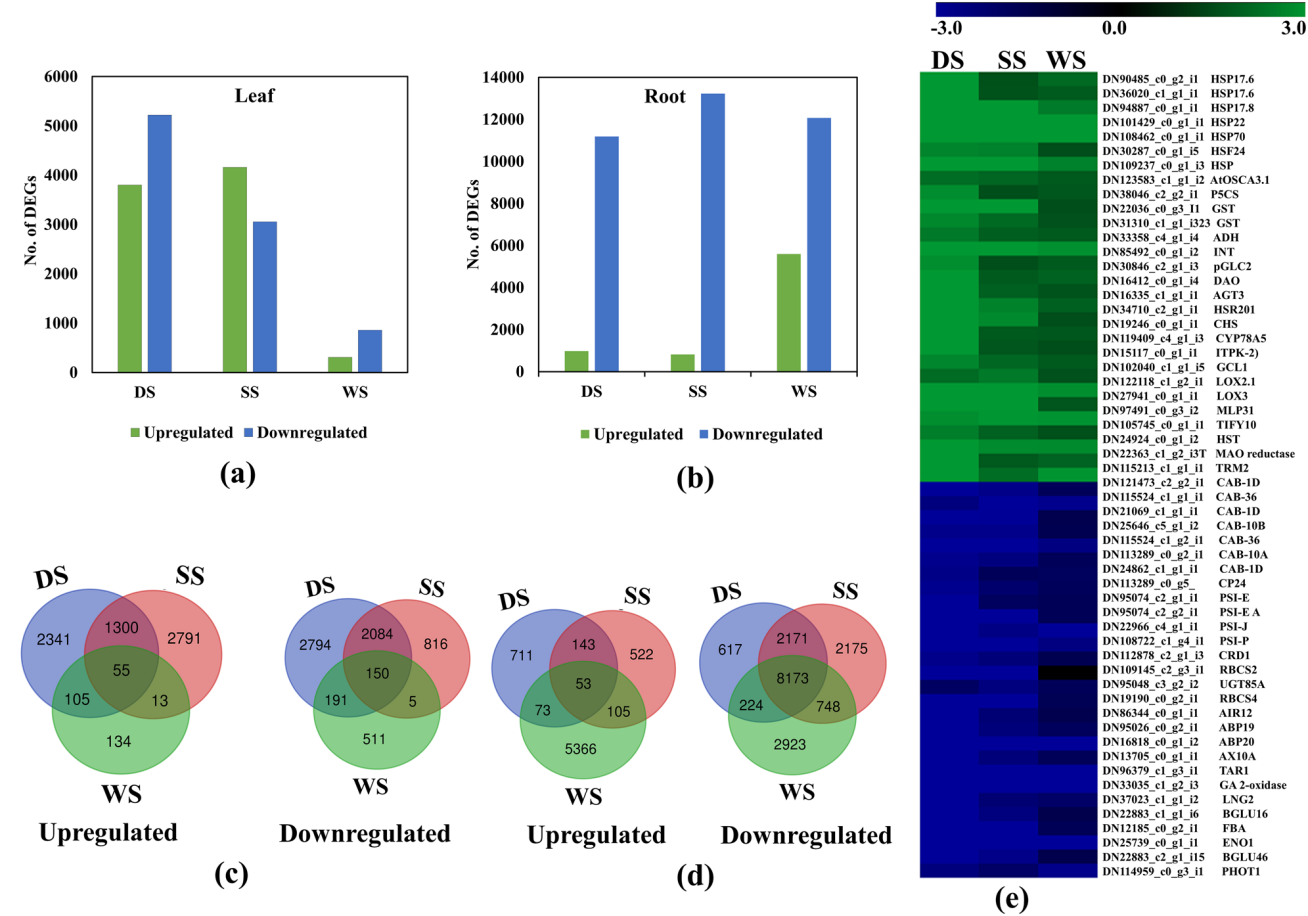
Deep transcriptome sequencing inferences of multiple abiotic stress responses under DS, SS, and WS in *S. rebaudiana*. (**a**) Dynamics of up and down-regulated DEGs under DS, SS, and WS in leaf &; (**b**) root tissue; (**c**) Vein diagram representing unique and common DEGs under DS, SS, WS in leaf & (**d**) roots; (**e**) Heatmap analysis of common DEGs expressed in leaf under three abiotic stresses.

Overall, 9020 (DS), 7214 (SS), and 1164 (WS) in leaf and 12,165 (DS), 14,049 (SS), and 17,665 (WS) transcripts in root were exhibited with a significant differential expression (fold change, log FC ≥ 1) in a pairwise comparison between treated and control samples (Fig. 3a,b; Supplementary data 2). Further, reference-based comparative analysis of differentially expressed genes (DEGs) identified significant DEGs including 6714 (DS), 4066 (SS), and 750(WS) in the leaf, and 2891(DS), 4011(SS), and 1949 (WS) in the root (Supplementary data 2). Less abundance of DEGs in the reference-based analysis may be attributed to incomplete annotations and/or differences in exon-level expression^24^. Therefore, *de-novo* assembly with genome IDs was used for downstream analysis. Among the three abiotic stresses, DS was recorded with a higher abundance of DEGs followed by SS and WS in the leaf (Fig. 3e). Comparatively, a higher abundance of downregulated DEGs (DS, SS, and WS) was recorded in roots (Fig. 3b). Interestingly, 205 DEGs (55 up-regulated, 150 down-regulated) in leaf, and 8226 DEGs (53 up-regulated, 8173 down-regulated) in roots were shared in all three abiotic stresses including DS, SS, and WS (Fig. 3c,d).

Functional characterization of common DEGs in three abiotic stresses (DS, SS, WS) in leaf and root tissue were identified (Fig. 3e). In leaf, upregulated expression of Heat shock proteins 17 family (HSPs 17.6, HSP17.8) probably modulating photosynthesis and ABA mediated signalling pathway; Glutathione S-transferases (GSTs) and Pyrroline-5-carboxylate synthase (P5CS) providing protection against oxidative damage and maintaining cellular homeostasis; OSCA3.1 a hyperosmolarity gated Ca2 + ion channel regulating stomatal movement and increasing water use efficiency under abiotic stress^25–28^. While, common DEGs encoding for the growth determining primary metabolism processes such as photosynthesis including photosystem I and II, Chlorophyll a-b binding proteins, rubisco subunits; Auxin (ABP199, ABP20) and Gibberellic acid (GA2-oxidase) were found downregulated. Similarly, in root 53 common upregulated DEGs encoding major categories included Myb-related protein, Expansin-A4, Basic leucine zipper 9, Gibberellin-regulated protein 1, and 3-oxo-Delta (4,5)-steroid 5-beta-reductase. While, 8173 shared downregulated DEGs in DS, SS, and WS in roots were encoded to growth (cellulose synthase), transporters (ABC transporter, Aquaporin, V-ATPase, Cation/H ( +) antiporter), and stress (HSP, Delta-1-pyrroline-5-carboxylate synthase) (Supplementary data 3).

### GO and KEGG pathway enrichment analysis

Gene ontology (GO) enrichment analysis with functionally annotated DEGs (*P*-value ≤ 0.05, FC ≥ 1) resulted into significant enrichment under DS (620 GO terms in leaf; up: 359, down: 262; 666 GO terms in roots; up:187, down:479), SS (580 GO terms in leaf, up: 384, down:196; 301 GO terms in root, up:24, down:277) and WS (121 GO terms in leaf, up:66, down:55; 1143 terms in roots, up:439, down:704) categorized into the biological process (BP), cellular component (CC) and molecular function (MF). Among the top 20 highly enriched GO terms associated with ‘photosynthesis and chlorophyll metabolism’ were identified under suppressed categories, whereas, ‘cellular response to stress’, ‘abiotic stimulus’, ‘lipid metabolism’, ‘protein misfolding’ and ‘terpenoid metabolic processes’ were upregulated under each abiotic stress in leaf (Figure S2). In the root, the GO terms associated with ‘ribonucleoprotein complex’, ‘DNA replication and protein biosynthesis’, and ‘NAD(P) + nucleosidase activity’ were highly enriched under DS, SS, and WS (Figure S2). There were only a few enriched GO terms shared among the three abiotic stresses in the root. The GO term unique to specific abiotic stress annotated to upregulated DEGs in leaf and root under DS, SS, and WS was given in Table ST3.

KEGG enrichment analysis with significant DEGs under DS, SS, and WS revealed enrichment of key biological pathways in the leaf including ‘carbon metabolism’, ‘photosynthesis’, ‘porphyrin and chlorophyll metabolism’ related to growth; ‘biosynthesis of secondary metabolites’ and ‘plant hormone signal transduction’, ‘MAPK signaling pathway plant’ were abundant in leaf (Figure S1b). Furthermore, photosynthesis (ko00195), chlorophyll (ko00860), carbon fixation (ko00710), and ‘plant hormone signal transduction’ (ko04075) exhibited with higher enrichment of key genes (DEGs fold change > 1) under DS and SS than WS. While, irrespective to DS, SS, and WS, root exhibited with enrichment of pathways encoding energy metabolism (pyruvate metabolism, glycolysis/gluconeogenesis oxidative phosphorylation), protein degradation (endocytosis, ubiquitin-mediated proteolysis), and ‘plant hormone signal transduction’ (Figure S1b).

### Abiotic stress-responsive transcription factors

The significant abundance of DEGs encoding TF genes were recorded both in leaf [3368 (DS); 2677 (SS); 305 (WS)] and root [(3133 (DS); 3767 (SS); 4834 (WS)] (Supplementary data 4). Among the various TFs, bHLH, NAC, MYB, C2H2, ERF, B3, and WRKY were most abundant and exhibited dynamic expression under DS, SS, and WS (Figure S3a; 3b). Wherein, bHLH, MYB, and NAC were recorded with upregulated expression in the leaf under SS. Interestingly, DEGs encoding TFs recorded with upregulated expression in the leaf (Up-25) and root (Up-24) were commonly shared among the three abiotic stresses i.e., DS, SS, and WS (Figure S3b). Among the shared upregulated TFs, an abundance of GATA, NF-Y, HSF, MYB, CAMTA, and Dof in the leaf, while ERF, MYB, and bZip in root suggest their tissue-specific expression preferences to activate abiotic stress response (Figure S2c). Likewise, stress-specific preferences were also evident with up-regulated DEGs under DS (WRKY70, DREB), SS(DREB), and WS (RAV1, WRKY54) in *S. rebaudiana* (Figure S4).

### Effect of abiotic stress on primary metabolism, stress pathway, and SGs biosynthesis

#### Photosynthesis and chlorophyll metabolism

**Figure 4:**
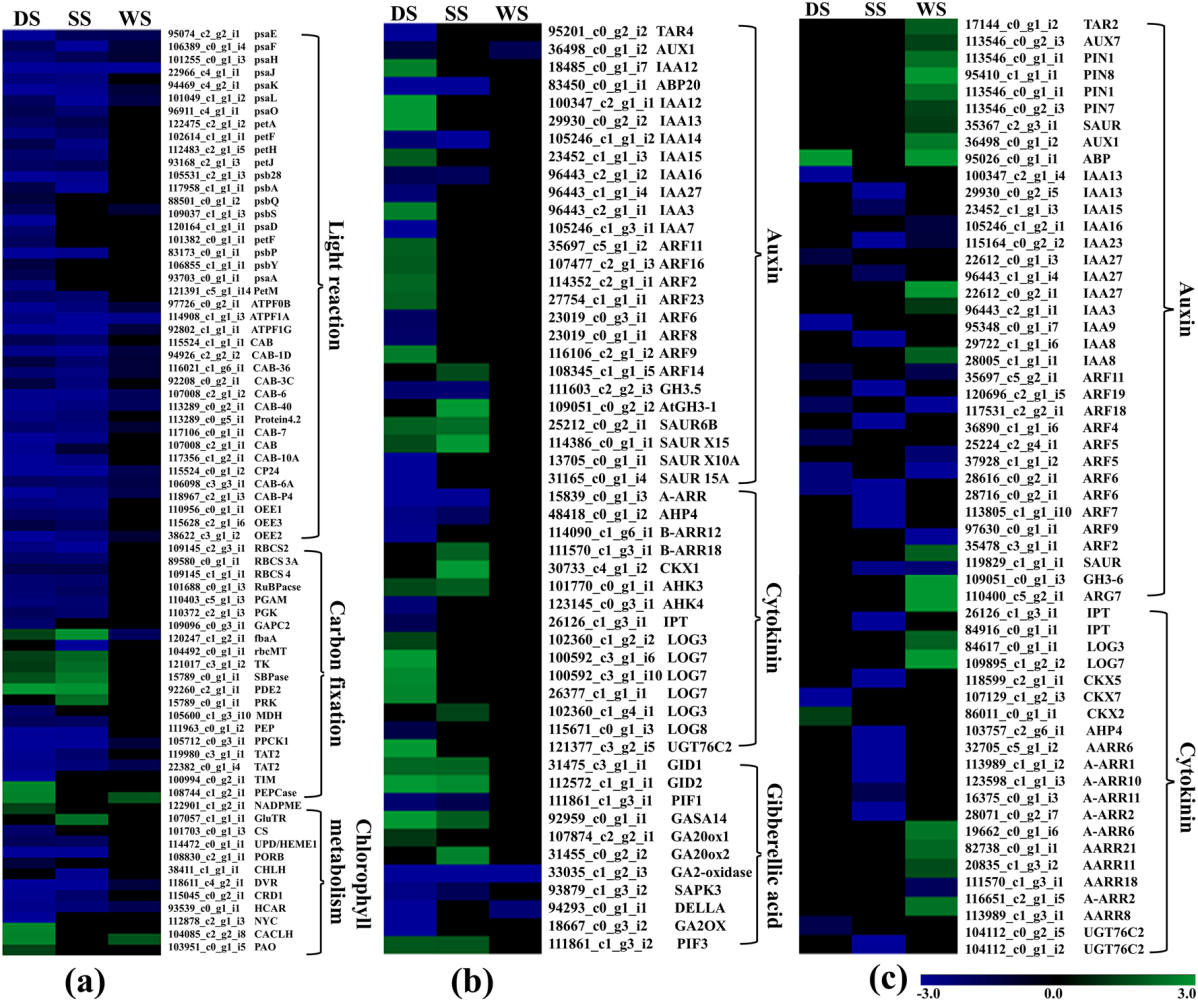
Heatmap analysis of differentially expressed genes related to primary metabolic processes under DS, SS, and WS. (**a**) Heatmap analysis of differentially expressed transcripts encoding for Photosynthesis and chlorophyll metabolism. (**b**) Heatmap analysis of differentially expressed transcripts encoding for plant growth hormones in leaf; and (**c**) root tissues.

Overall, DEGs regulating PSI & II and LHCI &II exhibited dynamic expression patterns under DS, SS, and WS (Fig. 4a). DEGs encoding light reaction were well complemented with the physiological decline in photosynthetic rate (Pn) under DS, SS, and WS. The important genes (PsbP and PsbA) of the oxygen-evolving complex (OEC) and D1 protein complex of LHCII were recorded with downregulated expression under DS and SS. Likewise, DEGs involved in the carbon fixation process (dark reaction) were recorded with downregulated expression under DS and WS. Furthermore, key DEGs regulating chlorophyll catabolism including NYC, CaCLH0, and Pao process under DS, while, GluTR and CHLH are involved in chlorophyll biosynthesis under SS recorded with upregulated expression (Fig. 4a). Nevertheless, only a few chlorophyll associated DEGs remained downregulated expression under WS.

#### Growth regulating plant hormone

The expression of DEGs encoding plant growth hormones (auxin, cytokinin, and gibberellic acid) biosynthesis and signaling were severely affected under three abiotic stresses. In the leaf, DEGs encoding for TAR4 and AUX1 transporters were significantly downregulated under DS with no significant expression under SS and WS (Fig. 4b). Additionally, DEGs encoding Auxin-responsive protein (AUX/IAA) family genes regulating cell signaling were upregulated under DS, whereas remained downregulated under SS. While auxin responsive DEGs including GH3.1 and SAUR gene family regulating plant growth were recorded with upregulated expression under SS. Furthermore, cytokinin receptors (AHK3 and AHK4) and cytokinin-mediated signaling processes (B-ARR12 and B-ARR18) exhibited dynamic expression under DS (downregulated) and SS (upregulated) (Fig. 4b). GA-associated DEGs showed downregulated expression under DS, while GA 20-oxidase regulating the active form of GA, and Gibberellin-regulated protein (GAST1 homolog) recorded with upregulated expression under SS (Fig. 4b). The DEGs encoding plant growth hormones including auxin, cytokinin, and GA in the leaf remained unaffected under WS. In the root, auxin transporters (PIN1, 7, and 8) support lateral root formation and cytokinin biosynthesis genes (IPT, LOGL3, and CKX7) exhibited with upregulated expression under WS, while DEGs involved in biosynthesis and signaling of auxin and cytokinin were downregulated under DS and SS (Fig. 4c).

**Figure 5:**
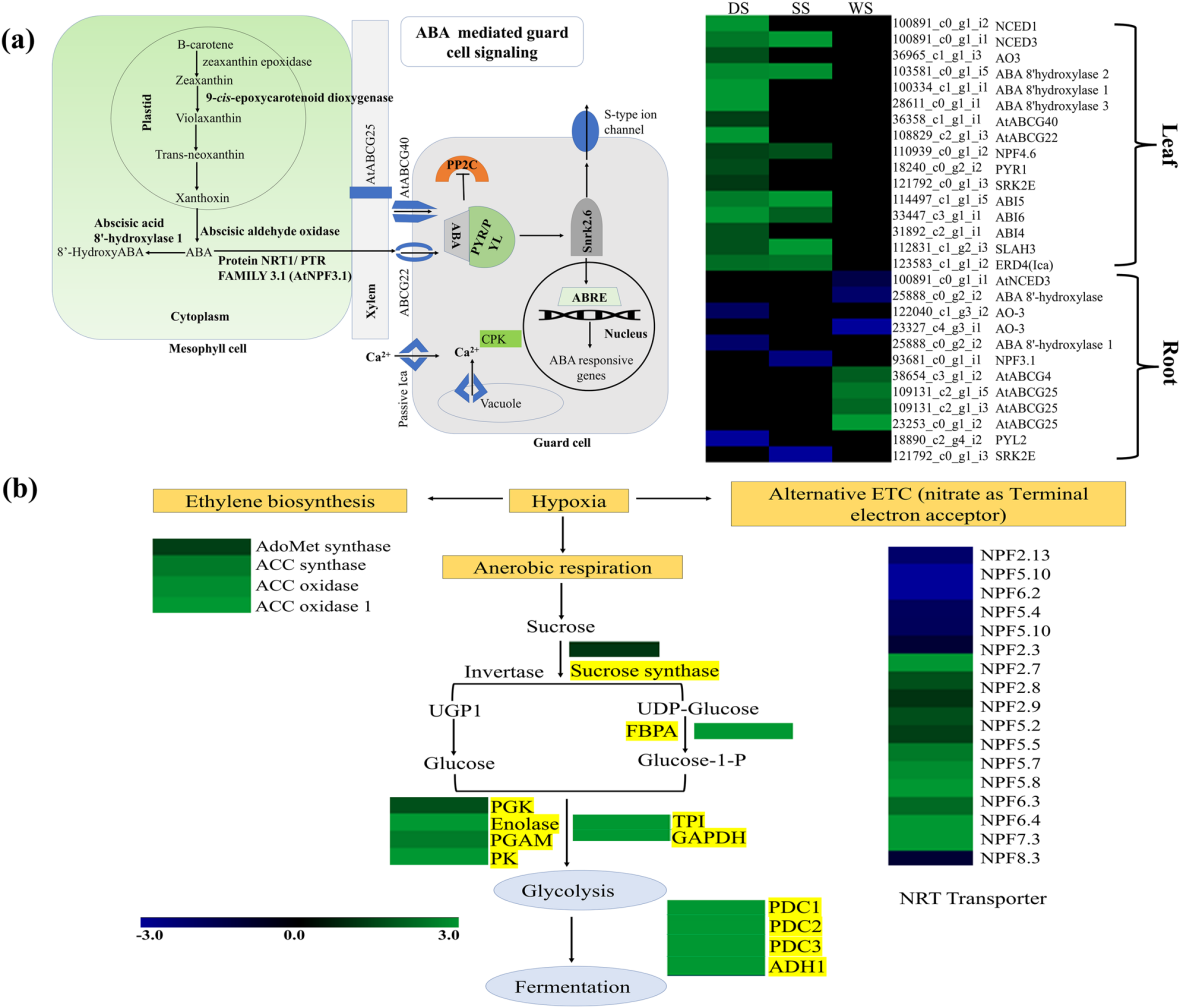
Abiotic stress-responsive pathways analyzed in this study. (**a**) Diagrammatic representation of ABA biosynthesis and induced signaling pathway and heatmap analysis of respective DEGs; (**b**) Hypoxia-mediated anaerobic respiration pathways and heatmap analysis of hypoxia-associated genes under WS in the root.

#### Abiotic stress-response plant hormones

ABA biosynthesis and ABA-mediated signaling pathway regulating transporters and ion channels were recorded with dynamic expression under all three tested abiotic stresses in *S. rebaudiana* (Fig. 5a). In the leaf, DEGs encoding for ABA biosynthesis (NCED1, 3, and AO3) and catabolism (ABA8'OH 1, 2, 3, 4) were upregulated under DS and SS (Fig. 5a). Additionally, transporters including ABCG22, ABCG40, and NPF4.6 facilitating the ABA uptake in guard cells and other vascular tissues were also exhibited with upregulated under DS, while only NPF4.6 recorded with upregulated expression in SS. Furthermore, the ABA-mediated core signaling pathway genes including PYR/PYL/PCAR (ABA receptor), PPC2, SnRK2s, and ABRE (ABRE-4,5), and downstream induced ion channels (SLAC1, and Ica) were significantly upregulated under DS, only a few in SS, while no significant expression was recorded under WS in leaf (Fig. 5a). All the ABA biosynthesis encoding DEGs were recorded with downregulated expression in the root (Fig. 5a). While, DEGs encoding ethylene biosynthesis including AdoMet synthase, ACC synthase, and ACC oxidase reportedly induced lateral root formation, and submergence adaptation, were recorded with upregulated expression in the root under WS (Fig. 5b).

**Figure 6:**
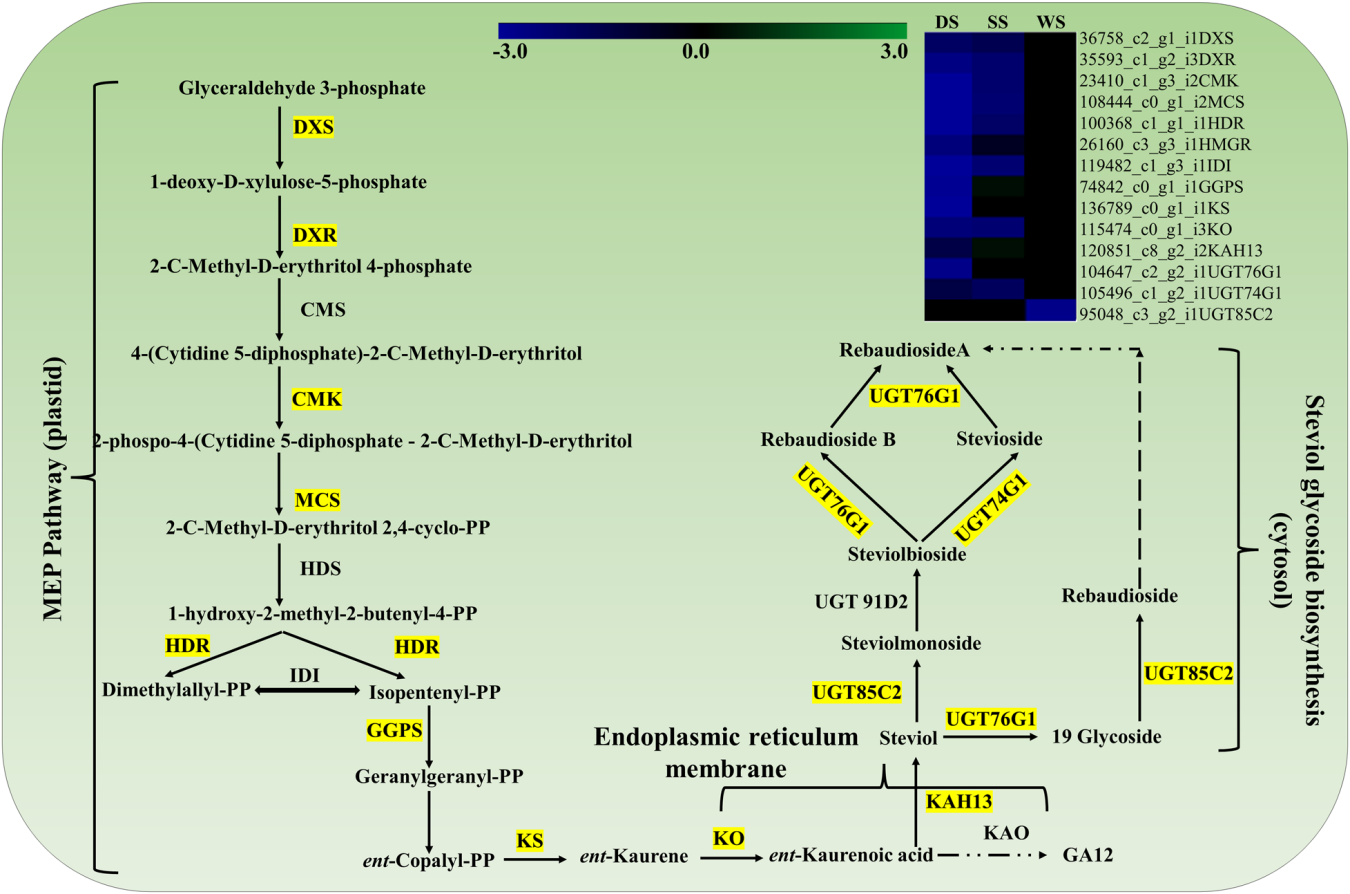
Steviol glycoside (SGs) biosynthesis pathway and the heatmap showing the expression profile of SGs pathway genes expressed under abiotic stress.

### Anaerobic pathway

Reduced oxygen supply to the cells during WS is reportedly involved in boosting the glycolysis process to compensate for energy requirement by producing ethanol as an end product, generating two molecules of ATP. DEGs encoding genes involved in fermentation including alcohol dehydrogenase (ADH), lactate dehydrogenase (LDH), pyruvate decarboxylase (PDA), and glycolysis pathway [sucrose synthase, aminotransferases (aspartate and alanine) generating glucose-1-P and pyruvate], respectively were recorded with upregulated expressions in the root under WS (Fig. 5b). Furthermore, 14 DEGs encoding nitrate transporters functioning as terminal electron acceptors in anaerobic conditions for the ATP synthesis were also found upregulated (Fig. 5b) ^29^. Overall, the current DEGs dynamics suggest that the anaerobic respiration processes work efficiently, possibly involved in reducing the severity of WS in the root.

### Abiotic stress-responsive kinases and phosphatases

DEGs encoding kinases and phosphatases including calcium-dependent protein kinases (CDPK), mitogen-activated protein kinase (MAPK), SNF-related kinase (SnRK), protein phosphatase (PP2C), calcineurin B-like protein (CBL), and calcium interacting protein kinase (CIPK) involved in stress signaling pathways recorded with dynamic expression in leaf [87 DEGs: DS (51), SS (33), WS (3)] and root [96 DEGs: (DS (32), SS (54), WS (59)] (Supplementary data 5). Most of the kinases, CBL, and phosphatases exhibited significantly upregulated expression in SS and were largely (except CIPKs) recorded with downregulated expression in DS. CDPK28 and MAPKs were uniquely expressed in the leaf under WS. The upregulated expression of CBL-CIPKs (SOS pathway) possibly indicates a defense mechanism toward ionic stress in SS. Interestingly, CIPK23 was reported to regulate stomatal movement (ABA-independent), and K + transport exhibited upregulated expression under DS^30^. Likewise, PLDα1 regulating stomatal movement both in ABA-dependent and independent manner was recorded upregulated expression under DS^31,32^. Furthermore, MAPK1 was recorded with upregulated (SS) and downregulated (DS and WS) expression, possibly regulating the enzymatic activities of antioxidants inducing stress response in *S. rebaudiana*. In the root, DEGs encoding intermediate signal transduction genes (145 DEGs) including CIPK, PPC2, and CDPK gene families exhibited upregulated expression under DS and WS, while recorded with downregulated expression under SS in this study (Supplementary data 5).

### Osmotic and ion stress-responsive genes

DEGs induced downstream abiotic stress signaling pathways including Ca^2+^ transporter (CAX), K^+^ transporter (TPK, KEA2, HKT1, and HKT2), vacuolar proton pump (V-ATPase), vacuolar H( +)-pyrophosphatase (VPpase), cyclic nucleotide-gated ion channel (CNGS20), ligand-gated ion channel (GLR2.8), aquaporins (PIPs, TIPs), antioxidants (SOD, GR, and GS, POD) and osmolytes (P5CS) were abundant in leaf and root (Table 1). In leaf, DEGs encoding CAX, VPPase, and GLRs regulating electrochemical gradient and cellular homeostasis via Ca^2+^ and H + transportation recorded with upregulated (SS) and downregulated (DS) expression^33^. Likewise, dynamic expression of Na^+^/K^+^ antiporters exhibited with downregulated HKT1(DS), upregulated HKT2 (SS), and with their insignificant expression under WS suggests stress-specific DEGs preferences. In roots, DEGs encoding transporters including NHE, TPK, KEA2, Vppase, and V-ATPase recorded with downregulated expression under DS and SS, while, HKT1, CAX2, NHE, and V-ATPase recorded with upregulated expression in WS (Table1). Two aquaporin subfamily genes including TIPs (Tonoplast intrinsic protein) and PIPs (Plasma mebrane aquaporin) were recorded with a highly upregulated expression in both leaf and root tissues (Table 1). However, no significant expression was recorded under WS in the leaf and SS in the root. Similarly, DEGs encoding antioxidants (SOD, GR, GS) were recorded with highly upregulated expression in the leaf under DS and SS, while no significant expression was found under WS (Table 1). Contrarily, roots exhibited down-regulated expression of all the DEGs encoding antioxidant genes under SS and DS, while upregulated expression in WS (Table 1).

**Table 1:** Details of potential differentially expressed genes (DEGs) identified in the deep transcriptional analysis under three abiotic stress responses (DS, SS, WS) in *S. rebaudiana.*

Categories	DS_L		SS_L		WS_L		DS_R		SS_R		WS_R	
	UP	DOWN	UP	DOWN	UP	DOWN	UP	DOWN	UP	DOWN	UP	DOWN
Detoxification												
*Glutathione reductase*	4	–	4	–	–	–	–	–	–	2	1	2
*Superoxide dismutase*	1	2	1	2	–	–	1	1	–	5	10	–
*Catalase*	1	3	–	4	–	–	1	–	–	–	–	5
*Cationic peroxidase*	–	2	–	1	–	–	–	5	1	1	6	–
*L–ascorbate peroxidase*	2	1	–	4	–	–	–	–	–	6	–	3
*Peroxidase*	1	1	1	1	–	–	–	39	–	6	–	–
*Phospholipid hydroperoxide glutathione peroxidase*	1	2	2	1	–	–	–	–	–	–	–	–
*glutathione S-transferase*	4	–	4	–	–	–	1	5	2	1	–	–
*Catalase-peroxidase*	–	–	–	–	–	–	–	–	–	–	1	2
Osmotic adjustment												
*Aldehyde dehydrogenase family 7 member (ALDH7A1)*	3	–	–	–	–	–	–	–	–	–	–	–
*Arginine decarboxylase (ADC)*	1	–	–	–	–	–	–	–	–	–	–	–
*Delta-1-pyrroline-5-carboxylate synthase(P5CSs)*	9	1	–	–	–	–	2	–	–	2	2	4
*Alpha,alpha-trehalose-phosphate synthase (TPSs)*	4	–	6	–	–	–	2	–	–	6	1	10
*S-adenosylmethionine decarboxylase proenzyme (*	4	1	4	–	–	–	–	–	–	–	–	–
*Galactinol synthase*	7	–	3	–	–	–	–	–	–	–	–	–
*Late embryogenesis abundant protein*	2	–	9	2	–	–	–	–	–	–	–	–
*Dehydrin*	4	–	3	4	–	–	–	–	–	–	–	–
Water channel												
*Aquaporin NIP2-1*	–	7	1	1	–	–	–	1	–	–	4	5
*Aquaporin PIP1*	20	2	11	–	–	3	18	1	2	–	17	4
*Aquaporin SIP1*	–	1	–	–	–	–	–	–	1	–	–	–
*Aquaporin TIP*	2	7	1	3	1	3	7	4	3	–	8	3
Ion transporters												
*Cation transporter HKT1*	–	1	1	–	–	–	–	1	–	–	1	–
*Cation transporter HKT2*	1	–	–	1	–	–	–	–	–	–	–	–
*Vacuolar cation/proton exchanger (CAX)*	1	4	4	–	–	–	–	4	–	–	1	1
*Sodium/hydrogen exchanger (NHX)*	2	–	1	–	–	–	–	4	–	3	2	3
*Two–pore potassium channel (TPK)*	–	2	1	3	–	–	–	–	–	1	–	2
*K (* +*) efflux antiporter*	–	7	–	1	–	–	–	–	–	10	–	–
*Cyclic nucleotide–gated ion channel (CNGS)*	2	3	1	–	–	–	3	–	–	5	–	–
*Glutamate receptor 2.8(GLRs)*	1	2	3	–	–	–	–	7	–	–	–	6
*Vacuolar H(* +*)-pyrophosphatase (VPPase)*	3	10	3	–	–	–	2	–	–	2	1	3
*Calcium permeable stress-gated cation channel (CSC)*	–	1	–	–	–	–	–	–	–	–	–	–
*V-type proton ATPase (VATPase)*	1	–	1	–	–	–	1	32	–	3	2	10
*Phospholipase D alpha1(PLD)*	3	–	–	–	–	–	–	–	–	–	–	5
*Calcium uniporter protein*	–	–	1	–	–	–	–	–	–	–	–	–

### Abiotic stress influence on steviol glycosides (SGs) biosynthesis

The cross-talk between plant response to various abiotic stresses and secondary metabolites biosynthesis is critical for the accumulation of bioactive compounds. Significant DEGs encoding major genes including diterpenoid backbone (MEP), GA biosynthesis, and key SG biosynthesis UGTs (UGT85C2, UGT76G1, UGT74G1) exhibited with dynamic expression under DS, SS, and WS (Fig. 6). Among the three abiotic stresses, DEGs encoding major SG biosynthesis genes except UGT85C2 recorded no/ in-significant expression under WS. Interestingly, KAH13 involved in the bifurcation of SGs and GA biosynthesis, and GGPS regulating GA biosynthesis was recorded with upregulated expression under SS. However, key SG biosynthesis genes were recorded with downregulated expression under DS.

### Abiotic stress-associated protein–protein interactome network (PPIN) analysis

**Figure 7:**
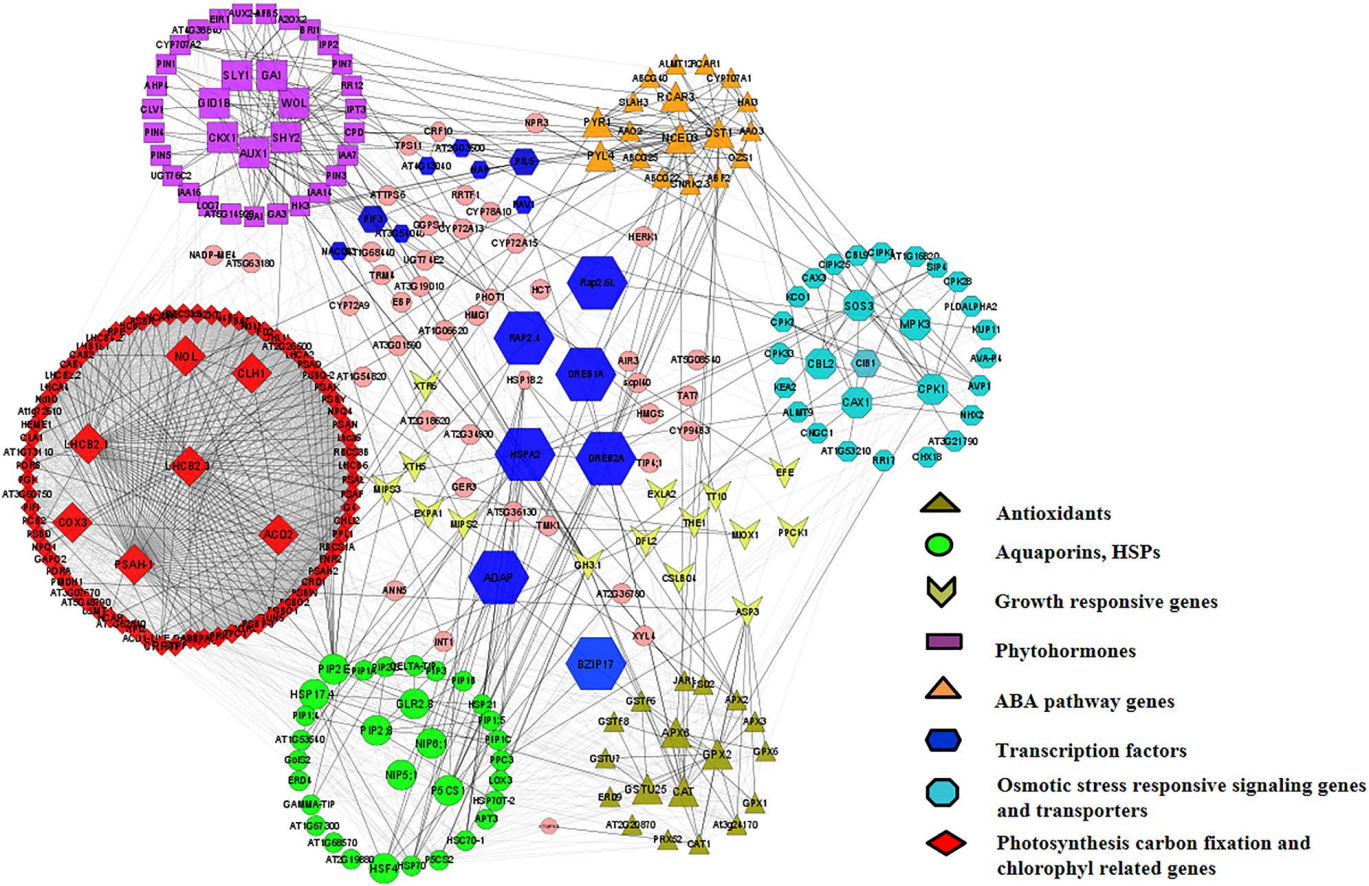
Interactome network analysis of DEGs in all three abiotic stresses; The significant DEGs were analyzed using STRING and visualized using CytoScape. Each node represents one gene and the edge represents protein interaction.

The PPIN analysis of significant DEGs encoding key genes and regulators activating abiotic stress response (DS, SS, and WS) and growth-related pathways resulted in 282 nodes and 2495 interactions (average neighbors: 17.695; clustering coefficient t: 0.223) (Fig. 7; ST 5). The overall network comprised 6 functional modules involving key DEGs regulating primary metabolism (modules 1 & 2), hormonal biosynthesis (modules 2 & 6), abiotic stress-responsive plant signaling & pathways (module 3, 4 & 5). Among these, module 1 was recorded with maximum interactions (≥ 50 interactions) representing key genes involved in photosynthesis (PHOT1, CAB, and LHCB) and chlorophyll metabolism (PORC, PORB). Module 2 (AUX1 ≥ 45 interactions, HK3, GA1) and module 6 (NCED3 ≥ 25 interactions) represent hormonal biosynthesis, and were enriched with key genes involved in growth hormones (PIN3, SHY2, GAI, IPT3) and ABA bio-synthesis (PYR1, ABF2, PYL4). The key abiotic responsive pathways (modules 3, 4 & 5) are enriched with osmotic and antioxidants (CAT, P5CS2), aquaporins (PIP), chaperones (HSP17, 70,18), and signalling molecules (kinases, transporters). Interestingly, transcription factors (DREAB2A, HSFA2, and PIF3) identified as major regulators in the predicted network showed significant interactions with each module.

### DEG validation by quantitative real-time PCR (RT-qPCR)

**Figure 8:**
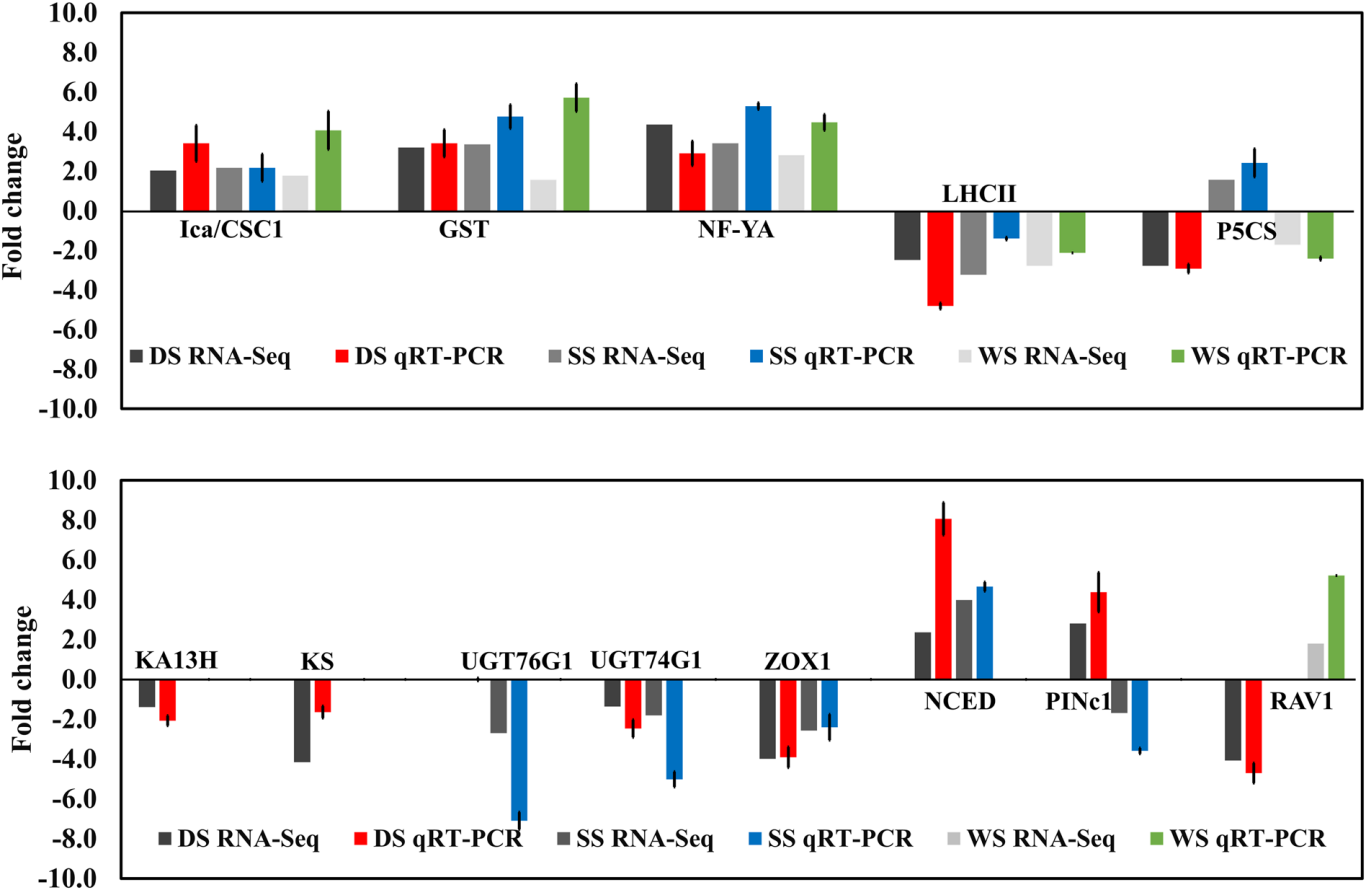
qRT-PCR validation for selected key significant DEGs shared in three abiotic stresses (DS, SS & WS). The columns indicate the qRT-PCR value of DS (Red), SS (blue), WS (green), and relative RNA_seq value (grey). Each bar represents the mean ± SD of triplicate assays.

To validate the DEGs, 14 significant DEGs encoding key genes involved in the activation of the abiotic stress response (DS, SS, WS), growth-related primary metabolism, and SGs biosynthesis in transcriptome data were chosen for qRT-PCR analysis. The qRT-PCR expression profile of five key DEGs including upregulated Ica/CSC1 (transporter), GST (antioxidant), NF-YA (transcription factor), and downregulated LHCII (Photosynthetic complex) under three abiotic stress conditions followed a similar trend with transcriptional data (Fig. 8). Other nine genes expressed in either one or two stress conditions, such as, SGs biosynthesis pathway genes exhibited downregulated expression under drought (KA13H, KS, and UGT74G1), and salt stress (UGT76G1, UGT74G1) showed good correlation (R^2^ = 0.6–0.7) with the transcription results (Fig. 8). Further key genes (GST, Ica/CSC1, NCED) utilized for the qRT expression analysis across the multiple genotypes treated with abiotic stress have complemented the expression patterns in compression with control under tested abiotic stressors (Figure S5).

## Discussion

The abiotic stresses, being a fundamental component of climate change greatly influence plant growth and productivity. The growth pattern of the plant species is consistently challenged by various abiotic stresses mediated by adverse environmental conditions caused by extreme drought, heat, cold, salt, and water^10^. The physiological and biochemical evidences have shown the significant negative impact of the abiotic stresses (drought, salt, waterlogging) on biomass, yield, and overall SGs accumulation in *S. rebaudiana*^20^. To enhance abiotic stress resilience, it is essential to gain a deeper understanding of gene regulatory networks of abiotic stress response for the development of climate-resilient high-yielding Stevia cultivars with optimum SGs accumulation. High-throughput next-generation RNA-seq analysis has been successfully applied to elucidate underlying molecular mechanisms of abiotic stress responses^34,35^. In the current study, for the first time, next-generation deep transcriptome sequencing, and morpho-physiological and biochemical analysis was implemented to enrich multiple abiotic stress-associated functional genomic resources, and elucidate the shared molecular mechanism/ gene regulatory network of abiotic stress response under drought (DS), salinity (SS), and waterlogging (WS) in *S. rebaudiana.*

### Abiotic stress-induced dynamics of physiological traits

The physiological attributes including SGM, photosynthesis, TCC, EL, and RWC were analysed to understand the impact of key abiotic stress (DS, SS, and WS) in *S. rebaudiana*. The severe (DS, WS) to moderate (SS) decline of plant growth might be associated with osmotic (DS, SS) or hypoxic (WS) conditions influencing primary metabolic activities such as cell cycle regulation and energy metabolism^36^. The growth responsive metabolism including photosynthesis process or gas exchange characteristics (Gs, Pn, and E) were impacted more due to limited water availability (DS) and absorption efficiency (SS) and (WS), and possibly contributed to declined plant growth, accordingly^37^. Similarly, a significant reduction of TCC, non-stomatal characteristics affecting light harvesting complex with more severity in DS than SS and WS were further add to declined photosynthesis process^38,39^. Additionally, severe change of both the abiotic stress responsive parameters indicating stress severity including cellular water content (RWC) and membrane stability (EL) affecting cellular homeostasis in DS, and minimum impact under WS, indicated the higher impact of former on Stevia^40^. Whereas, SS found with high EL which potentially indicate elevated Na^+^ and Cl^-^ ions accumulation was moderately impacted at physiological level. Overall, physiological parameters studied were more impacted under DS followed by SS and WS.

### RNAseq and DEGs analysis

RNAseq analysis has been successfully employed to elucidate the molecular insights and underlying mechanisms of abiotic stress response in several plants^41^. An integrated approach using references-based and de novo assembly resulted into 318,859 transcripts derived from more than 3 billion filtered reads suggesting deeper coverage of RNAseq data in the present study. A significantly high rate of mapping (83%) and annotations (71%) of NR transcripts with reference genome provided greater resolution of gene contents, organ-specific gene expression, and abiotic stress (DS, SS, WS) associated abundance of full-length key candidates of associated metabolic pathways^35,42^. Furthermore, consensus both in de novo and reference-based differential expression (DEGs) inferences identifying the same set of genes and regulatory candidates suggests the significance of current data. A variable abundance of DEG counts under abiotic stress conditions (DS, SS, WS) may be attributed to the severity and complexity of the abiotic stress type to *S. rebaudiana*^41^. Also, the modulation in biological processes requires a rapid transcriptional changes to cope with the specific abiotic stress^43^. Additionally, tissue-specific variations in DEG counts under DS and SS with comparatively higher abundance of downregulated DEGs in root than leaf indicating the tissue-specific severity of the abiotic stress response as reported in earlier studies^44^. Nevertheless, a significantly higher abundance of upregulated DEGs in the root than in the leaf indicates the efficient adaptive mechanism in the root toward WS in *S. rebaudiana*. Furthermore, expression profiling of common genes suggests more impact of DS than SS and WS in this study after 30 days of treatment. Functional characterization of the common DEGs encoding genes involved in primary metabolic processes (photosynthesis, growth hormones, growth-responsive genes) exhibited downregulated expression, while stress-responsive genes recorded with upregulated expression. Overall expression pattern suggests that the abiotic stress-responsive adaptive mechanism is operating well via channelizing energy and significant reduction of plant growth-associated metabolic pathways.

### Transcription factors mediated abiotic stress response

TFs in plants have been reported to play a significant role in regulating the expression of several abiotic stress-responsive genes via binding to their corresponding cis-acting elementary sequences^42,45^. The major transcription family genes (bHLH, MYB, NAC ERF) identified in this study were also reported in *Artemisia annua* under abiotic stress, which may imply a significant role in regulating downstream stress-mediated signal transduction pathways^21^. Additionally, the common TF family genes under DS, SS, WS in the leaf (B3, GATA, NF-Y, HSF) and root (ERF, MYB) may provide tissue-specific regulation of abiotic stress-responsive pathways in *S. rebaudiana*. The functional role of NF-Y family genes (upregulated), in regulating plant developmental processes (chloroplast biogenesis, cell proliferation, photosynthesis) has been reported^46^. Likewise, several earlier studies reported the key role of DEGs encoding the AP2/ERF TF family gene in the regulation of growth, development, and stress response^47,48^. Interestingly, up-regulated expression of AP2/ERF family genes (DREB1A and DREB2A) possibly regulates proline and sugar accumulation providing ABA-dependent mediated tolerance under DS and SS^49,50^. Similarly, RAV1(WS) a negative regulator of plant growth and development, WRKY70 (DS), and WRKY54(WS) modulating osmotic stress tolerance were found upregulated^51,52^.

### Abiotic stresses impact genes of energy metabolism

The group of genes encoding for Chlorophyll a/b binding proteins (LHC), Ribulose bisphosphate carboxylase large chain (RBCL), and Cytochrome P450 involved in photosynthesis (primary source of energy production) were impacted due to abiotic stress in this study. Among the three abiotic stresses, the higher abundance of downregulated DEGs encoding PSI and PSII under DS explained the severe physiological decline of photosynthetic rate than two other stresses (SS, WS) were also reported in maize and tobacco^53,54^. Nevertheless, some upregulated photosynthesis-associated DEGs may be responsible for the lower decline of plant shoot growth under SS. Likewise, least number of affected DEGs may associated with less impact on gaseous exchange and photosynthesis process under WS, and potentially attributed to the greater plant adaptability as reported earlier in the abiotic stress-sensitive genotype in citrus^55^. Moreover, the severe plant growth decline under WS might attributed to anaerobic respiration in root to compensate energy crisis, an adaptability mechanism under WS^56^. Interestingly, upregulated expression of anaerobic respiration-associated genes including PDC (PDC1 and 2) and ADH possibly providing tolerance toward anoxia under prolonged WS conditions^57^. Thus, in this study, the dynamic transcriptional changes in relation to energy and growth metabolism were in coordination to the physiological decline in Stevia under abiotic stresses with severe impact of DS followed by SS and WS.

### Hormonal-mediated regulation of abiotic stress response

The plant response to the multiple stresses is regulated by the cross-talk between the phytohormone signaling processes. In this study, gene expression analysis of major growth-promoting (Auxin, cytokinin, and GA) and abiotic stress-responsive hormones (ABA and ethylene) were investigated to analyze the stress-induced modulation and their effect on plant growth and survivability. In our study, DEGs regulating auxin, cytokinin, and GA signaling network were largely downregulated in both tissues under abiotic stress, wherein under SS (leaf) some cytokinin (CRE, B-ARR) and GA-mediated signaling DEGs were upregulated that may be responsible for the differential growth response. Cytokinin being antagonistic to the ABA-induced metabolic processes may responsible for the lesser reduction of physiological parameters for SS^58,59^.

Furthermore, DEGs associated with ABA-mediated signaling and biosynthesis possibly regulating osmotic and water potential adjustment were upregulated under DS and SS in the leaf. However, downregulated expression of the ABA biosynthesis gene NCED in the root might promote the adventitious roots (ARs) formation under waterlogging stress^60^. Additionally, ethylene biosynthesis (ACC synthase, AdoMet synthase, ACC oxidase) related genes were significantly upregulated in roots under WS. Ethylene promotes the adventitious root formation by regulating the upregulated expression of auxin transporter genes (Aux1 and PIN) in response to waterlogging stress^61^.

### Intermediate signal transduction genes regulating abiotic stress response

Under unfavourable environmental conditions, abiotic stress signal perception activates signal transduction genes regulating downstream functional genes (osmolytes, transporters, antioxidants) to maintain cellular homeostasis in plants^62^. Interestingly, upregulated expression of putative stress sensors including CNGCs, a family of glutamate receptor-like (GLR), and cell wall proteins (expansins, glycoproteins) possibly activate downstream signaling pathways under DS and SS in the leaf^63,64^. Likewise, CDPKs activating various signal transduction pathways (ABA, ROS mediated pathways) in response to abiotic stresses and plant development were downregulated in DS, while upregulated in SS^65^. Similarly, the upregulation of SnRK2, a key cellular energy sensor possibly induces a response to ABA regulating stomatal opening under SS and DS in the leaf^66^. Furthermore, MAPK family genes regulating osmotic and ionic homeostasis in plants under abiotic stress were significantly upregulated under SS (MAPK1, MAPK3, MAPKK2 MAPKK5, MAPKKK3) suggesting the positive regulation under SS^67^. Likewise, the upregulated expression of MAP3K18 might be the key putative regulator of DS response as overexpression transgenic in Arabidopsis exhibited tolerance to drought stress^67^. Similarly, DEGs encoding the CIPKSs gene in roots possibly involved in hormone signaling mediated regulation of developmental processes might contribute to tissue-specific activation of stress response under DS in *S. rebaudiana*^68^.

### Osmolytes, antioxidants, and ion transporters activated under abiotic stress

Osmolyte accumulation is required to maintain cellular membrane integrity and functions in response to various abiotic stress^69^. In this study, DEGs related to osmolyte biosynthesis (ALDH7A1, P5CSs, ADC, TPSs, GOLSs) were positively expressed due to DS in leaf and root, and negatively expressed in root under SS and WS indicating the varying response of Stevia toward different stress in growth and adaptation. Similarly, antioxidants are the key indicators of response towards increased biosynthesis of ROS from metabolic activities, providing defense against oxidative damage and cell death during various abiotic stresses^70^. DEGs encoding antioxidant enzymes were negatively affected under DS and SS in the root than leaf, whereas the opposite response was noticed under WS in this study. The upregulated DEGs encoding GSTs and GRs under DS and SS in the leaf, and SOD under WS in the root might be providing a tissue-specific abiotic stress response in *S. rebaudiana*. Likewise, upregulated expression of LEA and dehydrin might regulate cell protection under SS and DS in the leaf.

Transporters induced downstream of ionic/osmotic stress signaling are known to maintain cellular homeostasis during various abiotic stresses^71^. The Ca^2+^, an important stress indicators accumulated primarily under a variety of environmental stimuli inducing ion-channels (potassium and sodium)^72^. Upregulated expression of DEGs associated with Ca transporters (CAX, CNGS, and GLRs) in the leaf suggests positive regulation under SS and DS. However, the downregulated GLRs and CAX in root tissue indicate the negative response under WS, wherein upregulated CNGS under DS suggests a role in stress adaptation. Likewise, HKT1, an important Na^+^specific transporter, regulating the accumulation of extra sodium ions in leaf tissue was upregulated in SS and DS^73^. Additionally, significantly upregulated expression of NHX, an important Na^+^/H^+^exchanger regulated by the CBL/CIPK signaling pathway required for compartmentalization of sodium ions from the cytoplasm to the vacuole and TPK important K^+^channels maintaining the Na^+^/K^+^ratio possibly provide tolerance under SS^74^. Moreover, upregulated expression of DEGs encoding key aquaporin protein family (PIP, TIP, NIP, and SIP) might facilitate the water movement passively on the plasma membrane and tonoplast membranes in the leaf (DS and SS) and root (DS and WS), having a key role in maintaining growth and other primary metabolic activity (photosynthesis) under various abiotic stresses^75,76^.

### Prediction of functional gene modules regulating abiotic stress response

Gene network analysis was performed to find an association among primary metabolic processes and stress-responsive pathways genes in this study. In addition, it described the putative interactions between TFs and the targeted downstream genes of different pathways. In photosynthesis genes containing modules PHOT1, CAB, PORC, PORB, and LHCB had a high interactions among themselves, and were downregulated under abiotic stress might be responsible for the reduced plant growth. Further, the growth-determining hub genes of module 2 (AUX1, HK3, GA1) expressed differentially in DS, SS, and WS were found to interact with ABA biosynthesis (NCED3), chloroplast development (CLA1) and stress-responsive gene (TPSII) indicating the cross-talk among plant hormones which has been reported to be important for the plant development and activation of stress response. Likewise, three important upregulated TFs (ADAP, HSFA2, DREB) were found to be interacting with a group of stress-responsive functional genes such as chaperones (HSP17, 70,18), osmolytes (P5CS, GolS2), aquaporin (TIP and PIP) of module 3 working down stream of stress-mediated signaling may be further utilized for the abiotic stress tolerance in Stevia^75^. Interestingly, the DREB was found to be interacting with the ABA-associated upregulated pathway genes (NCED, PYR1, PYL4) and OST1 in DS, working downstream of ABA signaling that control stomatal aperture might working well under DS. Moreover, the interaction of DREB2A with intermediate signaling gene (SOS3) regulating transporters (CAX1, NHX2) indicates the transcriptional regulation of ionic stress expressed positively under SS in this study.

### Steviol glycoside biosynthesis and gene expression under abiotic stress

Steviol glycosides (SGs), a group of secondary metabolites derived from the diterpenoid pathway are majorly accumulated in leaf. The change in SGs accumulation and accompanied downstream gene expression varies depending on the type of abiotic stress suggesting the stress-specific adaptive responses under DS, SS &WS. Furthermore, upregulated KAH13 possibly be associated with the shift of GAs synthesis flux towards SGs synthesis as a result of which less decline was observed under SS^2^. The downregulated expression of both key UGTs (UGT74G1, UGT76G1) involved in the biosynthesis of stevioside and rebaudioside influencing SGs composition (RebA/Stev ratio) under DS and can be further explored for the developing of climate-resilient Stevia genotypes with optimal SGs accumulation. Overall, DEGs encoding major SG pathway genes with comparatively less impacted under WS following moderate impact in SS to severe in DS indicating varying impact on signaling processes involved in the SGs production and can be further evaluated across the plant development stages and in multiple genotypes for deeper understanding.

## Conclusion

The first integrated abiotic stress-responsive functional genomic resource created here using tissue-specific morpho-physiological and global transcriptomic data will provide a comprehensive functional genomic database for deeper understanding DS, SS, and WS response in *S. rebaudiana*. The key transcription factors (HSFA2, DREB1A, DREB2A) with coordinated expression and interactions with energy metabolism (photosynthesis, chlorophyll metabolism), stress-responsive key pathways (ABA, ethylene, ion stress) and functional genes encoding transporters (HKT, CAX, TPK), detoxification (SOD, catalase), osmotic regulators (P5CS, TPS1), (GST, HKT1, MAPKs, P5CSs, PIP) can be potential candidates to understand their mechanistic role in abiotic stress-mediated upscaling of SGs biosynthesis. Furthermore, significant interactions of HSP17.4 with HSFA2 can be a potential candidates for mitigating abiotic stress resilience. These potential candidate genes can be further extrapolated for genetic manipulation and targeted gene editing. The current findings could be useful in developing strategies to enhance SG accumulation and have the potential to develop climate resilient cultivars in *S. rebaudiana*.

## Materials and methods

### Plant materials and experimental setup

The experiment was conducted using micro-propagated rooted plants of superior cultivar ‘Him-Stevia (CSIR-IHBT-ST-01; National ID: IC0613966; Registration No. INGR15018, Registering Agency: Plant Germplasm Registration Committee (PGRC), Indian Council of Agricultural Research (ICAR), Govt. of India) developed by CSIR-Institute of Himalayan Bioresource Technology, Palampur, Himachal Pradesh, India (20.5937° N, 78.9629° E). Phenotypically uniform plants were transferred to each pot (diameter=10 cm, height=20 cm) filled with the soil: sand mixture (1:1 ratio), and watered once a day with half-strength of Hoagland’s liquid growth media (HiMedia Laboratories Pvt Ltd). The plants were first acclimatized in a growth chamber (Percival Scientific Inc) with photoperiod (16 h light-light flux-2,000–2,500 lx/8 h dark), temperature (24±1 °C), relative humidity (RH-70±5%) for four weeks before commencement of the experiment. Subsequently, plants were treated with three abiotic stresses viz. drought stress (DS), salinity stress (SS), and waterlogging stress (WS) treatments with three replicates per treatment for 30 days based on optimum response (duration/dose) in the initial screening (data not shown). The DS condition was imposed by water with 50% of field capacity (FC) with a one-day hold. SS condition was implemented by watering NaCl dissolve in 200 ml of water to achieve the 120 mM concentration. WS condition was created and maintained by filling the pots up to 2 cm above the root-shoot junction in the soil. The control samples were maintained under irrigation with 200 mL of simple water regularly once a day. Physiological data were analyzed before the collection of the tissues. For RNA isolation and transcriptome analysis, leaf and root were collected from each pot after 30 day of experiments and stored at−80°C.

### Plant growth analysis

The shoot growth measurement (SGM) was performed to analyze the plant's growth under DS, SS, and WS. To measure the shoot growth, the first node from the tip was noted with a white thread and the initial length (L1) was measured using a calibrated scale (cm) at the beginning of stress treatment (day ‘0’). The post-treatment shoot length (L2) was re-measured at the end of the experiment (day ‘30’) to record the effect of DS, SS, and WS on shoot extension^77^.

### Physiological analysis

Photosynthetic parameters including photosynthetic rate (Pn, mmol m-2 s-1), stomatal conductance (Gs, molm-2 s-1), and transpiration rate (E, mmol m-2 s-1) for fully expanded leaves were assessed with a portable infrared gas analyzer (LI-6400; LI-COR Inc., Lincoln, NE, USA)^71^. The assimilation chamber was maintained at 23 °C ± 1 °C, with RH of 50–60% ± 5% and CO2 concentration of 400 μmol CO2 mol^−1^. All observations were made at a photosynthetic photon flux density (PPFD) of 500–800 mol m2 s1 (light saturation point). The chlorophyll content was measured in mg/m^2^using a CCM-300 chlorophyll content meter (Opti-Sciences, Inc., Hudson, NH). An average of the content of a total of three mature leaves per plant was considered for chlorophyll measurement^78^. Similarly, the relative water content (RWC) of three fully expanded leaf samples per plant was evaluated^79^. Briefly, fresh leaf samples were weighed (FW) and put in distilled water for 24 h at room temperature in the dark. The leaf samples were then blotted dry and weighed to determine their turgid weight (TW). To calculate the dry weight (DW), the leaf samples were oven-dried at 70 °C. The RWC was calculated using the formula (FW–DW)/(TW–DW) ×100. To determine the electrolyte leakage (EL), fresh 100 mg leaf samples were collected in deionized water containing a 15 ml glass vial and oscillated at 1200 rpm for 12 h at room temperature. Initial electrical conductivity (EC1) was measured after 12 h of incubation using the Eutech PC 700 conductometers meter. The same samples were autoclaved for 1 h at 120 °C and final electrical conductivity (EC2) was measured. EL was calculated using the formula, EL (%) = (EC1/EC2) ×100^80^.

### Steviol glycosides (SGs) estimation

The leaf samples from the individual pot were collected, oven-dried (40±2 °C for overnight), and ground to make a fine powder^75^. 100 mg of dried leaf powder was used to prepare extract in 10 ml methanol for HPLC (Waters, Milford, MA, USA) analysis. Further HPLC analysis was performed using LiChrosphere® 100 NH2 column (250 mm×4 mm×5 m particles) (Merck, Germany) system with mobile phase Acetonitrile: Water (8:2). The sample was analyzed for 20 min and detected at a wavelength of 210 nm using commercial standards for Stev and Reb A, RebF, RebC, and DulA^81^.

### RNA extraction, library construction, and Illumina sequencing

Total RNA was extracted from treated (DS, SS, and WS) and control leaf and root samples in three biological replicates using the iRIS protocol^82^. RNA quantification and RNA IQ (integrity and quality) were analyzed using QubitTM RNA IQ Assay proto-col (Life Technologies, Thermo Fisher Scientific Inc). 4 µg total RNA for leaf and root samples from three replicates derived from DS, SS, and WS stress conditions were utilized to prepare sequencing libraries using the TruSeq Stranded mRNA library preparation kit (Illumina, San Diego, CA). Standard library size distribution and concentration were assessed using Bio-analyser Chip DNA 1000 Series II (Agilent Technologies USA). Libraries were sequenced over Illumina NovaSeq 6000 sequencing platform (Illumina, San Diego, CA) to obtain 200 bp paired-end reads.

### Sequencing data analysis and functional annotation

Raw reads obtained after Illumina sequencing were processed for high-quality data filtering using NGS QC Toolkit with Phred score≥30 (http://www.nipgr.res.in/ngsqctoolkit.html). De-novo assembly of high-quality reads was accomplished using Trinity version v2.8.2^83^with a 300 bp minimum transcript length. Further, clustering with 90% identity of these transcripts was performed using CD-HIT (v4.6.7)^84^. The assembled transcripts were functionally annotated using NCBIs NR, KEGG (Kyoto Encyclopedia of Genes and Genomes), TAIR10 (The Arabidopsis Information Resource), Swiss-Prot (http://www.expasy.ch/sprot), and Plant Transcription Factor Database (PlantTFDB) (http://planttfdb.cbi.pku.edu.cn/).

### Assessment of DEGs and pathway enrichment

The reads from each sample were aligned to de-novo assembled transcripts using the Bowtie2 aligner (http://bowtie-bio.sourceforge.net/bowtie2/index.html). Further transcript quantification in terms of FPKM was carried out using RSEM tool^85^. The differential gene expression (DGEs) of three abiotic stresses in comparison to control conditions of both leaf and root tissue was conducted using the edgeR tool considering *p*-value < 0.05 and log fold change (logFC) ≥1^80^. Further significant DEGs were subjected to GO enrichment analysis using the R package cluster profiler^86^. The KEGG enrichment of different biological pathways was performed using the KEGG database (http://kobas.cbi.pku.edu.cn/)). PlantTFDB (http://planttfdb.cbi.pku.edu.cn/) was utilized to identify stress-specific differentially expressed PTFs.

### Reference-guided transcriptome analysis

For reference-based data analysis, filtered reads obtained from treated (DS, SS, WS) and control (C) samples were mapped on the Stevia genome^87^using the hisat2 tool (http://daehwankimlab.github.io/hisat2/). Transcript abundance in terms of FPKM was performed using STRINGTIE TOOL v2.2.0 (https://ccb.jhu.edu/software/stringtie/). Differential gene expression analysis was analyzed with the help of edgR tool using*p*-value < 0.05 and log2FC≥1^88^.

### Interactome analysis of DEGs

The protein–protein interactome network (PPIN) analysis was used to obtain vital information on abiotic stress-mediated plant signaling and regulatory components^89^. Interaction among abiotic stress pathway genes and with other processes such as photosynthesis, growth hormones associated genes, and TF were determined using a string database (http://string-db.org/) and further visualized using Cytoscape_v3.8.2^90^.

### Validation of DEGs by quantitative real-time PCR (RT-qPCR)

The qRT-PCR validation was performed to validate the DEGs of key genes obtained from RNA-seq analysis. The specific primers of housekeeping (Actin) and targeted genes were designed using BatchPrimer3 (http://probes.pw.usda.gov/batchprimer3/) (Table ST4). The RNA was extracted from three biological replicates of treated and control samples, and cDNA was prepared using RevertAid H Minus First Strand cDNA Synthesis Kit (Thermo Scientific, Lithuania). The qRT-PCR reaction was set up using Power SYBR® Green PCR Master Mix (QuantStudio 5 Real-Time PCR Systems, Applied Biosystems USA). To quantify the expression of specific genes, the relative expression analysis was done using the 2− ΔΔCt quantification method^2^.

### Statistical analysis

Statistical analysis of the data of treated (DS, SS, WS) and control (C) samples collected in three biological replicates were performed using IBM SPSS Statistics 25 (SPSS Inc. Chicago, Illinois). The one-way ANOVA with Duncan’s multiple range test was applied to get the significance of the differences between the means (*p* < 0.05).

### Supplementary Information

Supplementary Information 1.

Supplementary Information 2.

Supplementary Information 3.

Supplementary Information 4.

Supplementary Information 5.

Supplementary Information 6.

## Data Availability

The raw sequencing reads were submitted to the Bioproject number PRJNA909184 Sequence Read Archive (SRA) of the National Centre for Biotechnology Information (NCBI) with accession number SRR22544232-SRR22544255.https://dataview.ncbi.nlm.nih.gov/object/PRJNA909184?reviewer=1t54rjbsc2b1hukfup9up239ub.
