# Supplementary material for: Genome-wide transcriptional profiling and physiological investigation elucidating the molecular mechanism of multiple abiotic stress response in Stevia rebaudiana Bertoni
Source: Sci Rep. 2023 Nov 13;13:19853. doi: 10.1038/s41598-023-46000-7 (PMC10645737; doi:10.1038/s41598-023-46000-7)

Research Paper

Title: Genome-wide transcriptional profiling and physiological investigation elucidating the molecular mechanism of multiple abiotic stress response in *Stevia rebaudiana* Bertoni

Poonam Pal[1,2], Mamta Masand[1,2], Shikha Sharma[1,2],Romit Seth[1], Gopal Singh[1,2] , Sanatsujat Singh[1], Ashok Kumar Yadav[1] ,Ram Kumar Sharma[1,2*]

[1]CSIR-Institute of Himalayan Bioresource Technology , Palampur -176061, India

[2]Academy of Scientific and Innovative Research (AcSIR), Ghaziabad-201002, India

***Correspondence:**
Dr. Ram Kumar Sharma
Biotechnology Division
CSIR-Institute of Himalayan Bioresource Technology
Palampur (H.P)
India
Email: rksharma.ihbt@gmail.com

Table ST1: Summary of Illumina generated sample-wise read information for 24 samples(raw read and filtered read) and mapping % of filtered reads on reference genome in *S. rebaudiana*. (C_L: Control_leaf; DS_L: Drought stress_leaf; SS_L: Salt stress_leaf; WS_L: Waterlogging stress_leaf; C_R: Control root; DS_R: Drought stress leaf; SS_R: Salt stress_root; WS_R: Waterlogging stress_root)

Sample detail	Raw reads	Filtered reads	Mapping% on the reference genome
C_L1	157208824	126844632	84.22%
C_L2	235205490	187570824	87.50%
C_L3	233621792	162834858	87.34%
DS_L1	179252334	139101150	84.24%
DS_L2	157111262	109811772	87.56%
DS_L3	158359182	126442772	86.02%
SS_L1	164293032	140056234	88.61%
SS_L2	156372390	125201916	87.26%
SS_L3	189000936	150060418	87.85%
WS_L1	214250770	146670206	88.17%
WS_L2	199847576	159675414	89.53%
WS_L3	163318122	134747470	84.84
C_R1	143765744	107512090	68.85%
C_R2	149920782	92979948	75.00%
C_R3	133804352	96784574	85.11%
DS_R1	125536220	111337902	87.24%
DS_R2	171486248	118066914	80.74%
DS_R3	188934556	135208036	79.99%
SS_R1	140451492	104335172	84.62%
SS_R2	161338114	106689310	85.11%
SS_R3	154342830	111977028	82.16%
WS_R1	162528914	130873462	75.05%
WS_R2	182677418	148969522	82.80%
WS_R3	209893032	162239074	78.19%
Total	4132521412	3135990698	83.66%

Table ST2: Summary of *de novo* assembly transcriptome of *S. rebaudiana*.

**TRINITY Assembly Statistics**	
No. of assembled transcripts	318859
Total length of transcript(bp)	274829027
Average sequence length (bp)	861
N50 length(bp)	1396
GC content	41.50%

Table ST3: List of unique abiotic stress-specific GO term processes enriched under drought stress (DS), salinity stress (SS) and waterlogging (WS)

GO term	Description	Hits	p-value
**GO terms under drought stress (DS) in leaf**			
**GO:0006749**	Glutathione metabolic process	26	0.003647
**GO:0009896**	Positive regulation of catabolic process	19	0.018135
**GO:0009687**	Abscisic acid metabolic process	13	0.017426
**GO:0004575**	Sucrose alpha-glucosidase activity	7	0.002712
**GO:0005987**	Sucrose catabolic process	5	0.018763
**GO:0071218**	Cellular response to misfolded protein	11	0.04302
**GO:0009963**	Positive regulation of flavonoid biosynthetic process	4	0.041248
**GO:0072665**	Protein localization to vacuole	11	0.020034
**GO:0043269**	Regulation of ion transport	13	0.028553
**GO:0015081**	Sodium ion transmembrane transporter activity	4	0.013339
**GO:0046345**	Abscisic acid catabolic process	4	0.000821
**GO:0046345**	Abscisic acid catabolic process	4	0.000821
**GO terms under drought stress (DS) in root**			
**GO:0004024**	Alcohol dehydrogenase activity, zinc-dependent	5	0.008174
**GO:0005372**	Water transmembrane transporter activity	21	0.000866
**GO:0015925**	galactosidase activity	10	0.049689
**GO:1905421**	Regulation of plant organ morphogenesis	8	0.026039
**GO:1902584**	Positive regulation of response to water deprivation	3	0.014925
**GO:0010133**	Proline catabolic process to glutamate	3	0.009575
**GO:0009739**	Response to gibberellin	27	0.016447
**GO:0004553**	Hydrolase activity, hydrolyzing O-glycosyl compounds	79	0.005714
**GO:0015145**	Monosaccharide transmembrane transporter activity	4	0.048927
**GO:0017014**	Protein nitrosylation	8	0.00126
**GO:0051762**	Sesquiterpene biosynthetic process	4	0.000947

**GO:0051762**	Sesquiterpene biosynthetic process	4	0.000947
**GO terms under salinity stress (SS) in leaf**			
**GO:0007165**	Signal transduction	16	0.036167
**GO:0022804**	Active transmembrane transporter activity	88	0.041043
**GO:0009755**	Hormone-mediated signaling pathway	104	0.000793
**GO:0008194**	UDP-glycosyltransferase activity	65	0.008685
**GO:0015298**	Solute:cation antiporter activity	10	0.026393
**GO:0015368**	Calcium:cation antiporter activity	5	0.023648
**GO:0004672**	Protein kinase activity	163	0.016796
**GO:0006817**	Phosphate ion transport	5	0.023152
**GO terms under salinity stress (SS) in root**			
**GO:0004326**	Tetrahydrofolylpolyglutamate synthase activity	3	0.031081
**GO:0006206**	Pyrimidine nucleobase metabolic process	4	0.013465
**GO:2000104**	Negative regulation of DNA-dependent DNA replication	5	0.019263
**GO:0005666**	RNA polymerase III complex	6	0.018018
**GO:0045595**	regulation of cell differentiation	11	0.041278
**GO:0042575**	DNA polymerase complex	7	0.003759
**GO:0034399**	Nuclear periphery	4	0.001346
**GO:0006801**	Superoxide metabolic process	6	0.046332
**GO terms under waterlogging stress (WS) in leaf**			
**GO:0046394**	Carboxylic acid biosynthetic process	36	0.038108
**GO:0016597**	Amino acid binding	3	0.006633
**GO:0005524**	ATP binding	18	0.012478
**GO:0140657**	ATP-dependent activity	17	0.000446
**GO:0043168**	Anion binding	60	0.046657
**GO:0140657**	ATP-dependent activity	17	0.000446
**GO:0016053**	Organic acid biosynthetic process	37	0.049448
**GO:0006544**	Glycine metabolic process	3	0.006633
**GO:0030554**	Adenyl nucleotide binding	20	0.007652
**GO terms under waterlogging stress (WS) in root**			

**GO:0051787**	Misfolded protein binding	17	0.016414
**GO:0034220**	Ion transmembrane transport	6	0.026483
**GO:0008308**	Voltage-gated anion channel activity	8	0.00437
**GO:0070469**	Respirasome	75	0.001814
**GO:0016614**	Oxidoreductase activity, acting on CH-OH	106	0.008426
**GO:0015986**	ATP synthesis coupled proton transport	18	0.000492
**GO:0071555**	Cell wall organization	73	0.003633
**GO:0016052**	Carbohydrate catabolic process	20	0.027918
**GO:0098655**	Cation transmembrane transport	118	0.005872

Table ST4. Detail of primers pairs used for validation using RT-qPCR

Transcript ID	Primer Sequence
TRINITY_DN123583_c1_g1_i2Fw	GCTGTTGTGGTTTTTACAAGTA
TRINITY_DN123583_c1_g1_i2Rw	CTAATCTCCCGTTCGTAATAAG
TRINITY_DN22036_c0_g3_i1Fw	AGCTATTTTTCAAGCCGTTAT
TRINITY_DN22036_c0_g3_i1Rw	AGATATGTGTGTGCGGTATGT
TRINITY_DN27941_c0_g1_i1Fw	TATCCTCCTGTGAGCAAACT
TRINITY_DN27941_c0_g1_i1Rw	ATGTCGTGGTAATCCAATGT
TRINITY_DN115524_c1_g1_i1Fw	GGCTTCTCAGGTTGTTTTAAT
TRINITY_DN115524_c1_g1_i1Rw	AAGTTCCTTAACCTTCAACTCA
TRINITY_DN38046_c2_g2_i1Fw	GGCTCTAAAACCTAGCAAGATA
TRINITY_DN38046_c2_g2_i1Rw	AGTAGAACACCTAAGGGACATG
TRINITY_DN120851_c8_g2_i2Fw	GTATGTTTCTAACCGAGGAAGA
TRINITY_DN120851_c8_g2_i2Rw	AGTTGCTCTTTTAACACCTTGT
TRINITY_DN136789_c0_g1_i1Fw	GGAATGTTGGGGAAGATCAA
TRINITY_DN136789_c0_g1_i1Rw	ACCAATGGGAGATGGTTGAC
TRINITY_DN104322_c0_g2_i2Fw	GATATGTGAGTGATGTTTGGAA
TRINITY_DN104322_c0_g2_i2Rw	TGAGGGATTTACCTTATCCTTA
TRINITY_DN25856_c0_g1_i2Fw	CAATGGACAGATCAAATTACAA
TRINITY_DN25856_c0_g1_i2Rw	CATCCTCCATAATCGTCTTAAT
TRINITY_DN118955_c1_g1_i3Fw	ACTGGGTTAAACGGGCCAATA
TRINITY_DN118955_c1_g1_i3Rw	ACGAGCAAAGTGTTTGAAGAGA
TRINITY_DN100891_c0_g1_i1Fw	CGGATTAAAAGCTAGAGAAAGA
TRINITY_DN100891_c0_g1_i1Rw	CTTGAATATGATCTGGGATTTT
TRINITY_DN113546_c0_g1_i1Fw	CTCAAGGAATTGTACCTTTTGT
TRINITY_DN113546_c0_g1_i1Rw	CACAAACCAAGAATGATGTAGT
TRINITY_DN114691_c0_g2_i1Fw	TGCTGAGAAAACACACATACA
TRINITY_DN114691_c0_g2_i1Rw	CGTCACTTGGGGTAACTG

Figure S1. Functional annotation of the *de novo* assembled transcripts (a) and KEGG enrichment of differentially expressed transcripts (DEGs) in leaf and root tissues (b) under abiotic stress.

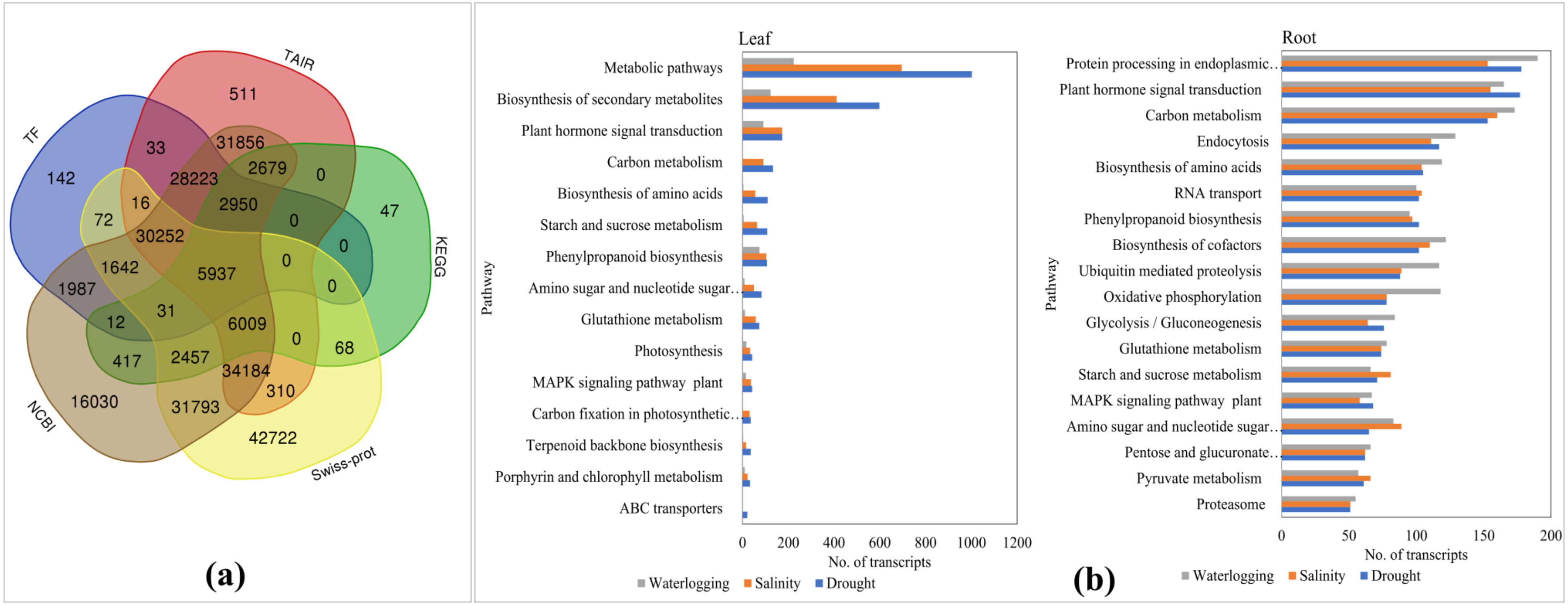

Figure S2. Gene ontology (GO) enrichment analysis. Dot plot shows GO terms upregulated and downregulated identified under **(a)** drought (DS_L and DS_R), **(b)** salinity (SS_L and SS_R), and waterlogging (WS_L and WS_R) stress using g:Profiler to be enriched (adjusted p-value < 0.05) among the genes. The size of the dots is based number of genes enriched in the pathway, and the color of the dots represents the adjusted p-values

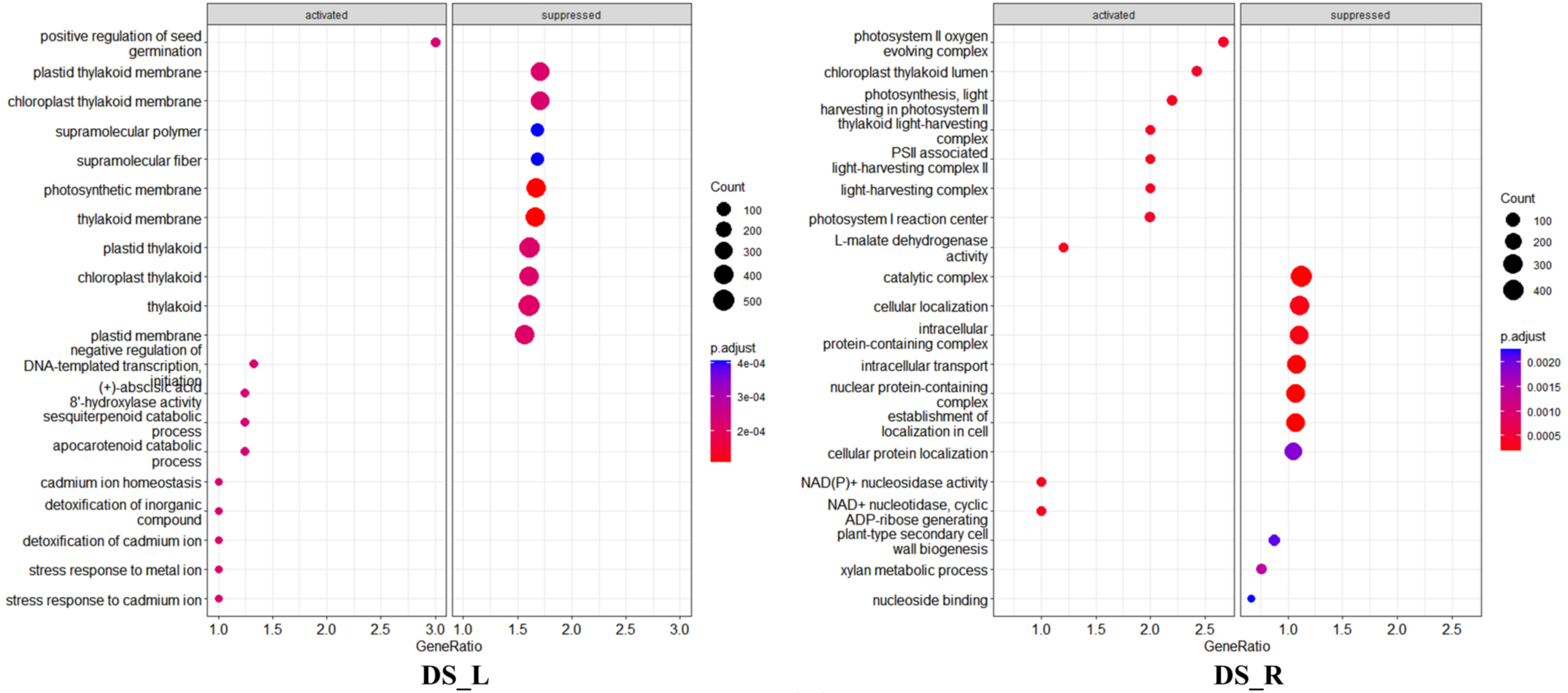

(a)

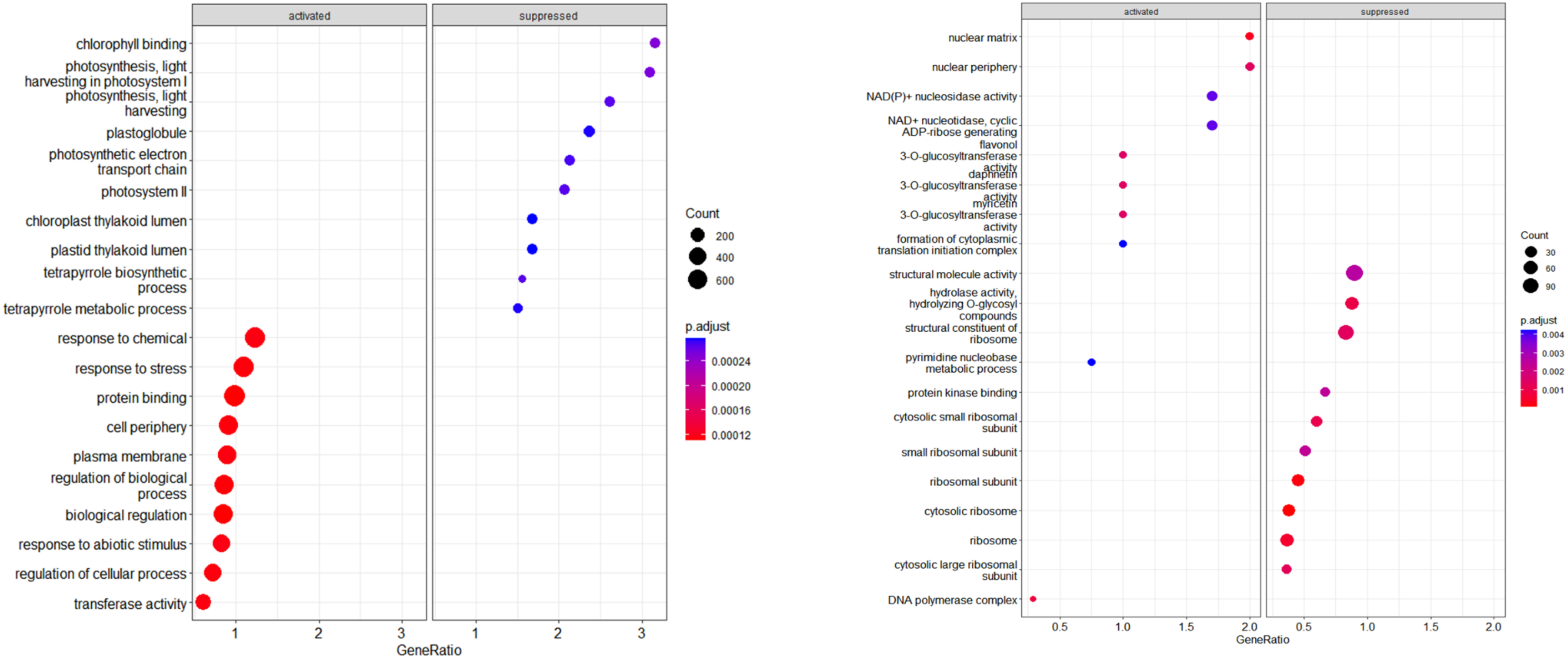
activated
suppressed
chlorophyll binding
photosynthesis, light harvesting in photosystem I
photosynthesis, light harvesting
plastoglobule
photosynthetic electron transport chain
photosystem II
chloroplast thylakoid lumen
plastid thylakoid lumen
tetrapyrrole biosynthetic process
tetrapyrrole metabolic process
response to chemical
response to stress
protein binding
cell periphery
plasma membrane
regulation of biological process
biological regulation
response to abiotic stimulus
regulation of cellular process
transferase activity
1
2
3
GeneRatio
Count
200
400
600
p.adjust
0.00024
0.00020
0.00016
0.00012
nuclear matrix
nuclear periphery
NAD(P)+ nucleosidase activity
NAD+ nucleotidase, cyclic ADP-ribose generating
flavonol 3-O-glucosyltransferase activity
daphnetin 3-O-glucosyltransferase activity
myricetin 3-O-glucosyltransferase activity
formation of cytoplasmic translation initiation complex
structural molecule activity
hydrolase activity, hydrolyzing O-glycosyl compounds
structural constituent of ribosome
pyrimidine nucleobase metabolic process
protein kinase binding
cytosolic small ribosomal subunit
small ribosomal subunit
ribosomal subunit
cytosolic ribosome
ribosome
cytosolic large ribosomal subunit
DNA polymerase complex
0.5
1.0
1.5
2.0
Count
30
60
90
p.adjust
0.004
0.003
0.002
0.001

SS_L **(b)** **SS_R**

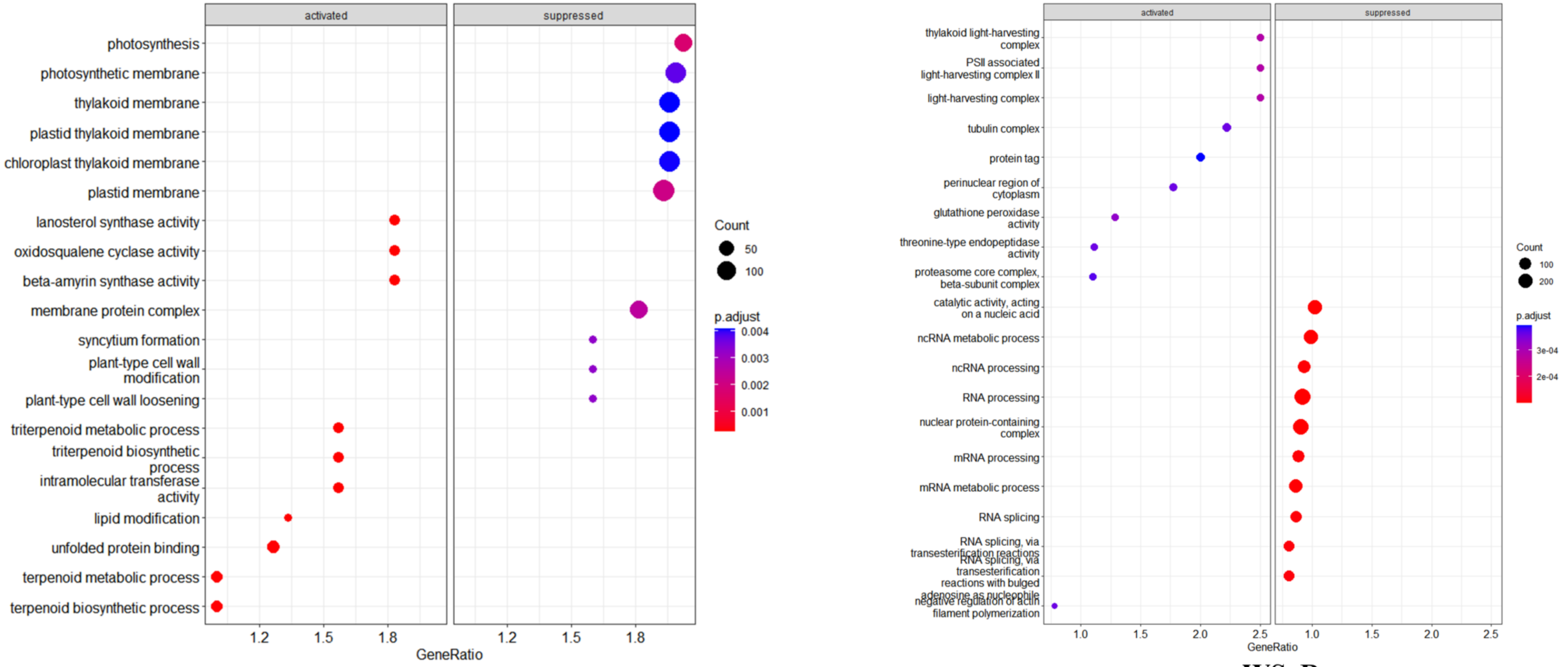
activated
suppressed
photosynthesis
photosynthetic membrane
thylakoid membrane
plastid thylakoid membrane
chloroplast thylakoid membrane
plastid membrane
lanosterol synthase activity
oxidosqualene cyclase activity
beta-amyrin synthase activity
membrane protein complex
syncytium formation
plant-type cell wall modification
plant-type cell wall loosening
triterpenoid metabolic process
triterpenoid biosynthetic process
intramolecular transferase activity
lipid modification
unfolded protein binding
terpenoid metabolic process
terpenoid biosynthetic process
1.2
1.5
1.8
GeneRatio
Count
50
100
p.adjust
0.004
0.003
0.002
0.001
thylakoid light-harvesting complex
PSII associated light-harvesting complex II
light-harvesting complex
tubulin complex
protein tag
perinuclear region of cytoplasm
glutathione peroxidase activity
threonine-type endopeptidase activity
proteasome core complex, beta-subunit complex
catalytic activity, acting on a nucleic acid
ncRNA metabolic process
ncRNA processing
RNA processing
nuclear protein-containing complex
mRNA processing
mRNA metabolic process
RNA splicing
RNA splicing, via transesterification reactions
RNA splicing, via transesterification reactions with bulged adenosine as nucleophile
negative regulation of actin filament polymerization
1.0
1.5
2.0
2.5
GeneRatio
Count
100
200
p.adjust
3e-04
2e-04

WS_L **(c)** **WS_R**

Figure S3. Differentially expressed transcriptional factor families under DS, SS and WS in *S. rebaudiana*. (a)Top 10 TF families expressed under DS, SS and WS. (b) The number of transcripts (up and downregulated) and respective Venn diagram representing shared (upregulated) TF among stresses in leaf and root tissues. (c) Heatmap analysis of differentially expressed shared TF encoding genes under DS,SS and WS.

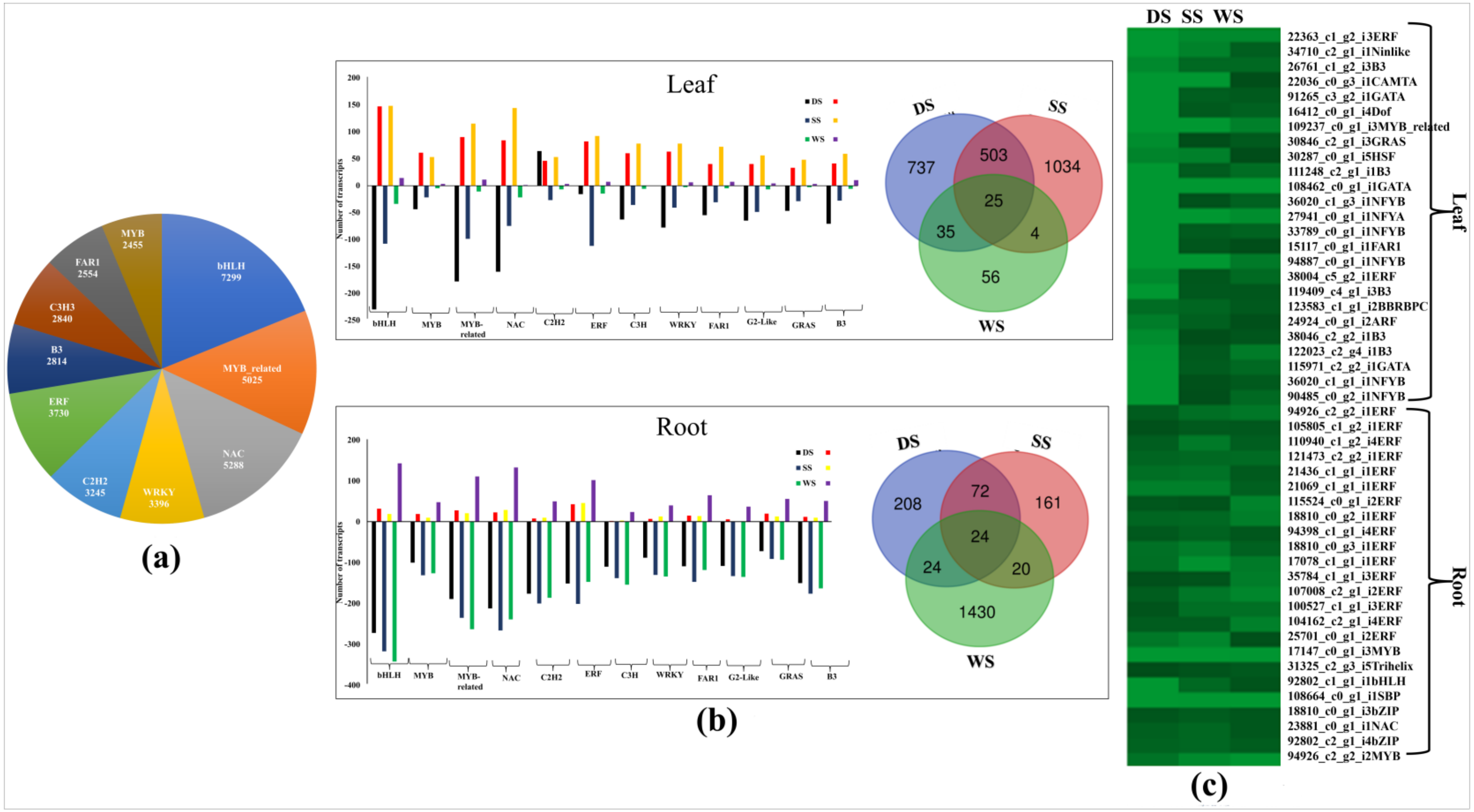

Figure S4. Heatmap analysis of differential upregulated transcripts encoding for TF unique to each abiotic stress in *S. rebaudiana*.

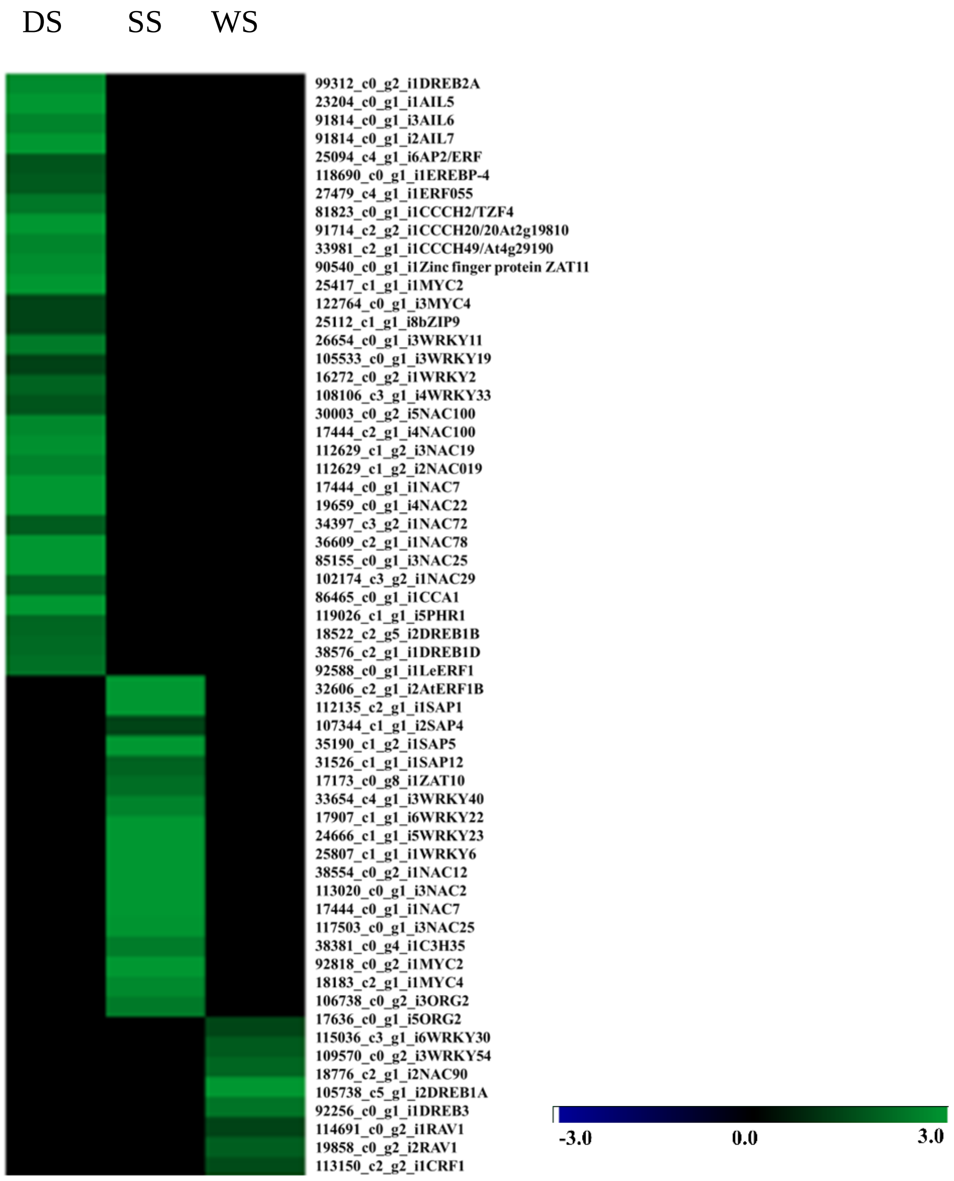

Figure S5. The qRT-expression analysis of the selected key significant DEGs in abiotic stress treatd multiple genotypes**.** The columns indicate the qRT-PCR value under DS (Red) and SS (green), in three genotypes. Each bar represents the mean ± SD of triplicate assays.

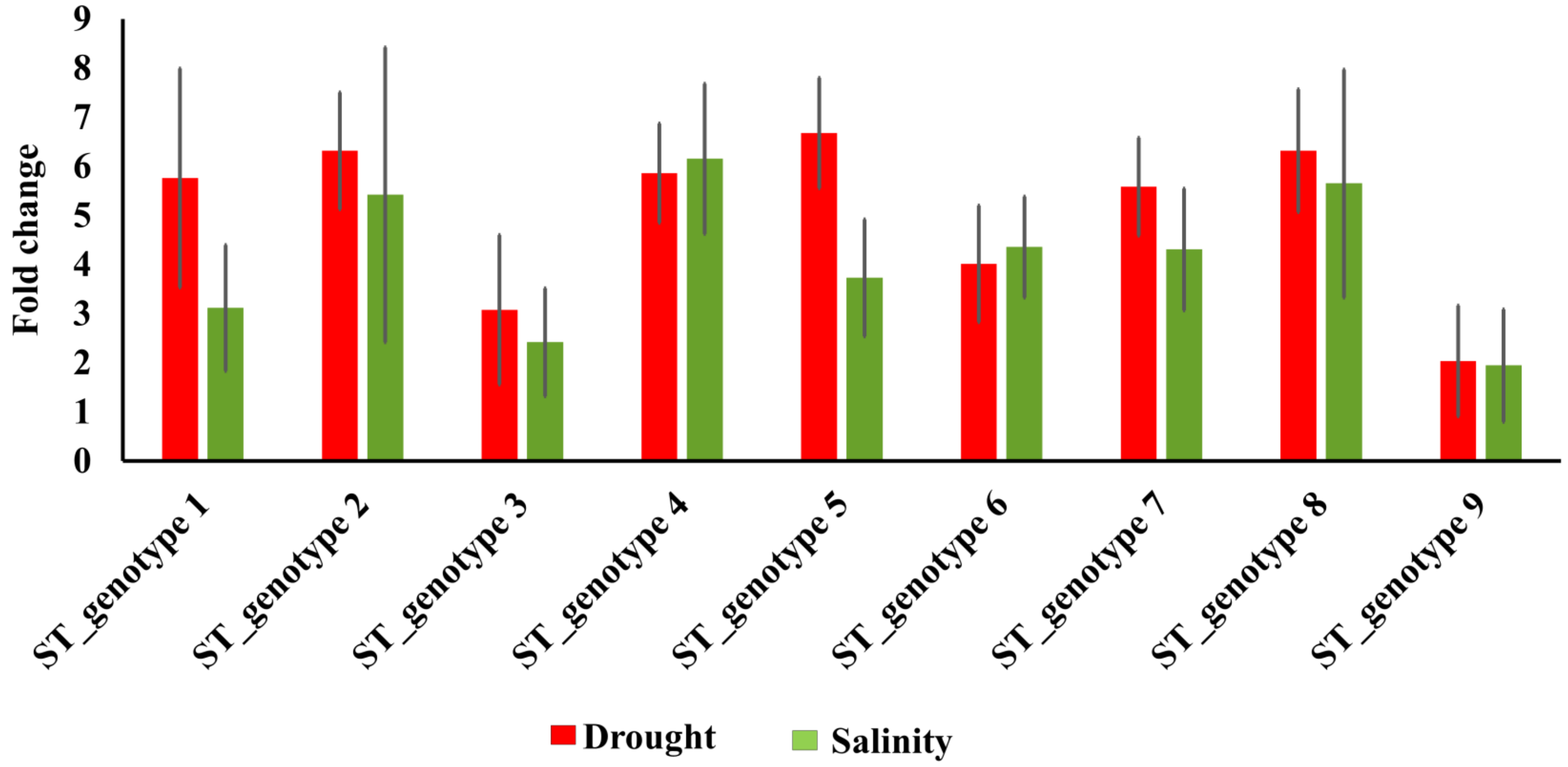

Supplement: Supplementary file 6 — Supplementary Information 6. [file 41598_2023_46000_MOESM6_ESM.pdf]
